# Blockade of C5a receptor unleashes tumor-associated macrophage antitumor response and enhances CXCL9-dependent CD8^+^ T cell activity

**DOI:** 10.1016/j.ymthe.2023.12.010

**Published:** 2023-12-14

**Authors:** Xiaojin Luan, Ting Lei, Jie Fang, Xue Liu, Huijia Fu, Yiran Li, Wei Chu, Peng Jiang, Chao Tong, Hongbo Qi, Yong Fu

**Affiliations:** 1 Chongqing Key Laboratory of Maternal and Fetal Medicine, The First Affiliated Hospital of Chongqing Medical University, Chongqing 400016, China; 2 Department of Obstetrics, The First Affiliated Hospital of Chongqing Medical University, Chongqing 400016, China; 3 Women and Children’s Hospital of Chongqing Medical University, Chongqing 401147, China; 4 Department of Gynecology, The Affiliated Hospital of Jiangsu University, Jiangsu University, Zhenjiang 212001, Jiangsu, China; 5 Department of Obstetrics, Yongchuan Hospital of Chongqing Medical University, Chongqing 402160, China; 6 Department of Reproductive Medicine Center, The First Affiliated Hospital of Chongqing Medical University, Chongqing 400016, China; 7 Department of Gynecology, The First Affiliated Hospital of Chongqing Medical University, Chongqing 400016, China

**Keywords:** complement C5a receptor, ovarian cancer, tumor-associated macrophages, T cell dysfunction, chemokine C-X-C motif ligand 9, immunotherapy

## Abstract

Macrophages play a crucial role in shaping the immune state within the tumor microenvironment (TME) and are often influenced by tumors to hinder antitumor immunity. However, the underlying mechanisms are still elusive. Here, we observed abnormal expression of complement 5a receptor (C5aR) in human ovarian cancer (OC), and identified high levels of C5aR expression on tumor-associated macrophages (TAMs), which led to the polarization of TAMs toward an immunosuppressive phenotype. C5aR knockout or inhibitor treatment restored TAM antitumor response and attenuated tumor progression. Mechanistically, C5aR deficiency reprogrammed macrophages from a protumor state to an antitumor state, associating with the upregulation of immune response and stimulation pathways, which in turn resulted in the enhanced antitumor response of cytotoxic T cells in a manner dependent on chemokine (C-X-C motif) ligand 9 (CXCL9). The pharmacological inhibition of C5aR also improved the efficacy of immune checkpoint blockade therapy. In patients, C5aR expression associated with CXCL9 production and infiltration of CD8^+^ T cells, and a high C5aR level predicted poor clinical outcomes and worse benefits from anti-PD-1 therapy. Thus, our study sheds light on the mechanisms underlying the modulation of TAM antitumor immune response by the C5a-C5aR axis and highlights the potential of targeting C5aR for clinical applications.

## Graphical abstract

**Figure:**
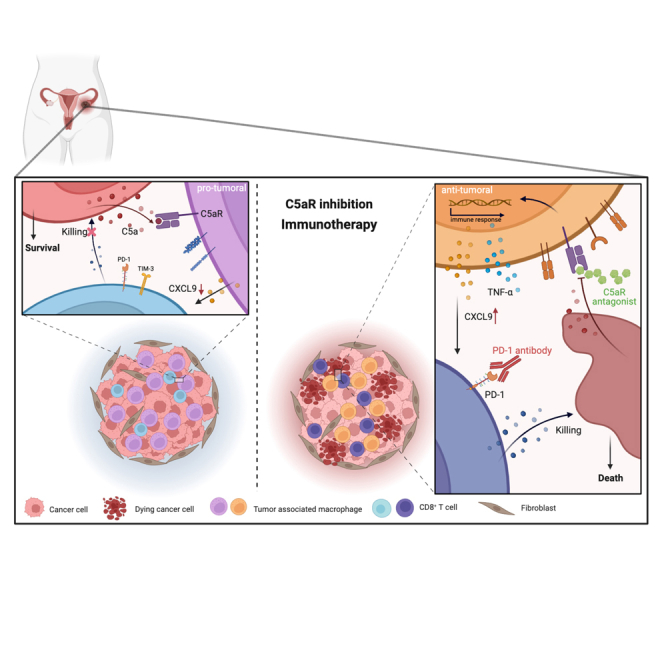

## Teaser

Fu and colleagues revealed that C5aR is a molecular switch controlling TAM antitumor activity, investigated the mechanisms underlying the modulation of TAM antitumor immune response by the C5a-C5aR axis, and proposed that the C5a-C5aR axis may serve as a potential biomarker for T cell dysfunction and the response to immunotherapy.

## Introduction

The complement system has been recognized as a significant component of innate immunity and is also considered to play a role in regulating adaptive immune response for immune surveillance and tissue homeostasis.^1^^,^^2^ Three distinct canonical pathways are involved in activating the complement system—classical, lectin, and alternative—and after activation, the complement system interacts closely with other components of innate immunity and cellular receptors (such as pattern recognition receptor and immunoglobulin G [IgG] Fc receptor) to participate in a series of immune responses, including clearance of target cells by membrane attack complex, opsonization of pathogenic microbes by C3b/iC3b, and activation and regulation of the inflammatory response by anaphylatoxins C3a/C5a.^3^^,^^4^

Beyond its classical function in innate and adaptive immunity, complement has long been considered to be essential for tumor immune surveillance. However, previous research has demonstrated that complement-derived mediators can also enhance the motility and invasiveness of human colon cancer cells through the abnormal expression of complement receptors.^5^ Moreover, accumulating evidence suggests that complement components have been extensively activated in multiple types of human cancer and play a detrimental role in antitumor immunity. Elevated complement levels in the blood or tumors have been found to contribute to tumor initiation and progression, and tightly correlate with poor clinical outcomes in cancer patients.^6^^,^^7^^,^^8^^,^^9^ Intratumoral C5a-C5aR signaling can induce recruitment of myeloid-derived suppressor cells (MDSCs) to the tumor microenvironment (TME), suppressing cytotoxic T cell response or dampening natural killer (NK) cell activation to promote tumor progression.^10^^,^^11^^,^^12^^,^^13^ Another report suggests that C5a may directly affect CD8^+^ T cell function by inhibiting interleukin-10 (IL-10)-dependent T cell-mediated antitumor immunity to promote tumor growth.^14^ Additionally, inhibiting complement components such as C3, C4, or C5aR by genetic knockout or using corresponding inhibitors has been shown to prevent tumor growth and metastasis.^11^^,^^12^^,^^15^^,^^16^

Macrophages constitute a major component of the immune infiltrate in the TME and play a crucial role in the host’s antitumor immunity.^17^ They have been shown to exhibit diverse functions in tumor progression, with some demonstrating an antitumor phenotype that possesses potent tumor-cell-killing ability, evidenced by enhanced phagocytosis or the mediation of T helper cells’ antitumor response. Conversely, macrophages with a protumor phenotype exhibit a compromised immune response and contribute to tumor progression.^18^^,^^19^ Previous research has demonstrated that cancer cells develop multiple evasion strategies to restrain host antitumor immune response by manipulating macrophage activity to form an immunosuppressive microenvironment. Tumor-associated macrophage (TAM) abundance has been found to increase in many human cancers, and increasing evidence indicates a functional intersection between tumor progression and macrophage immune status.^20^^,^^21^^,^^22^ Therefore, exploring the molecular switch that controls macrophage phenotype conversion might contribute to cancer therapy.

Numerous studies have demonstrated that the C5a-C5aR axis functions to impact macrophage antitumor immunity. In a model of squamous carcinogenesis, C5a regulated mast cells and macrophages expressing C5aR1^+^ to exhibit a protumorigenic property, resulting in promoted tumor progression.^23^ In addition, previous studies have reported that C5a contributes to M2-like TAM infiltration and secretion of proteases to promote metastasis of colon cancer cells.^24^^,^^25^ However, the exact mechanisms of how C5aR regulate TAM immune status and whether macrophage C5aR impacts the immune infiltrates and TME have not been fully determined. Thus, in the present study, by combined analysis of human and mouse ovarian cancer (OC) samples, we defined C5aR as a molecular switch that controls TAM antitumor activity and investigated its underlying mechanisms; in addition, we evaluated the promising role of C5aR blockade in cancer immunotherapy.

## Results

### High expression of C5aR is correlated with macrophage immunosuppression

**Figure 1:**
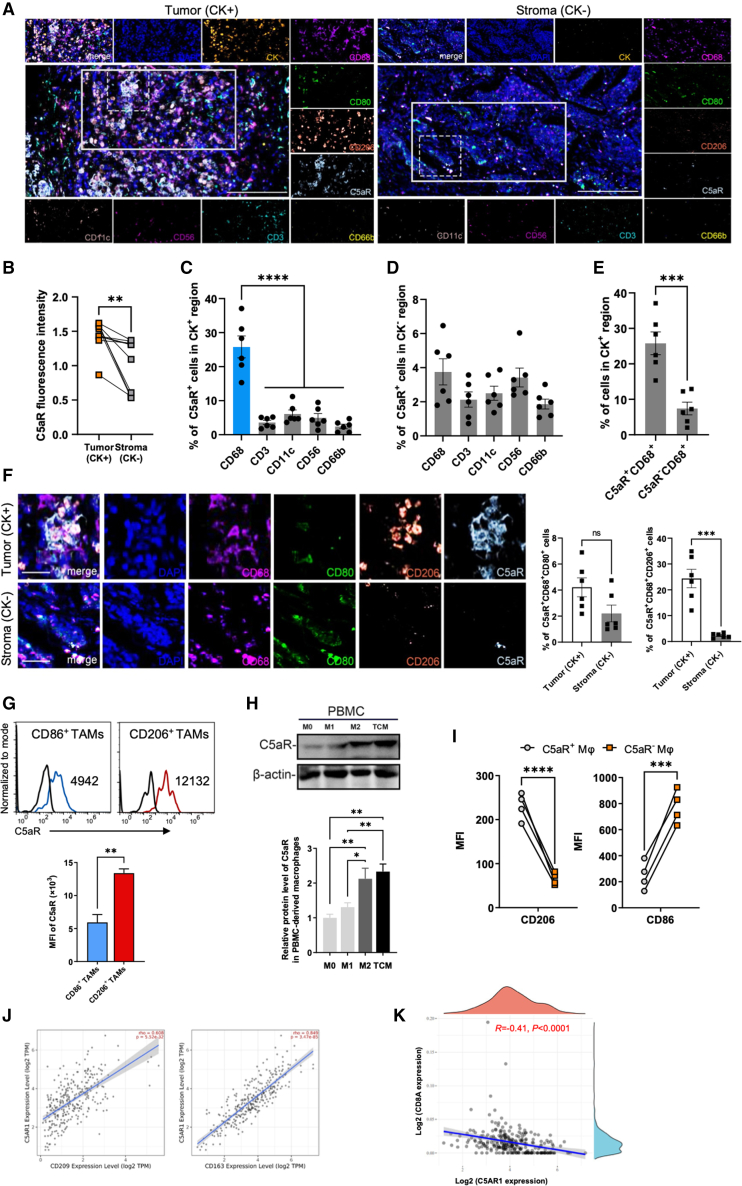
Tumor-associated macrophages highly express C5aR (A) Images of representative multiple immunofluorescence (mIF) results. Ovarian tumor (CK^+^) mIF image on left, ovarian stroma (CK^−^) mIF image on right. Scale bars, 100 μm. (B) Comparison of fluorescence intensity between tumoral and stromal C5aR^+^ cell (n = 6 samples; two-tailed paired t test). (C and D) Quantification of C5aR^+^CD68^+^, C5aR^+^CD3^+^, C5aR^+^CD11c^+^, C5aR^+^CD56^+^, and C5aR^+^CD66^+^ cells in CK^+^ or CK^−^ region of human OC tissues (n = 6 samples; one-way ANOVA). (E) Quantification of tumoral C5aR^+^ and C5aR^−^ macrophages (n = 6 samples; two-tailed paired t test). (F) Representative images and quantification of C5aR^+^CD68^+^CD80^+^ and C5aR^+^CD68^+^CD206^+^ cells in CK^+^ or CK^−^ region of human OC tissues (n = 6 samples; two-tailed paired t test). Scale bars, 25 μm. (G) Flow-cytometry analysis of C5aR expression on CD86^+^ TAMs (CD45^+^CD11b^+^Ly6G^−^F4/80^+^CD86^+^) or CD206^+^ TAMs (CD45^+^CD11b^+^Ly6G^−^F4/80^+^CD206^+^) in ID8 tumors (n = 3 mice; two-tailed paired t test; representative of three independent experiments). (H) Representative western blot analysis of C5aR expression in PBMC-derived macrophages receiving PBS (M0), LPS (M1), IL-4 (M2), and TCM treatment. β-Actin was used as a loading control (n = 3 independent replicates; one-way ANOVA). (I) Flow-cytometry analysis of CD206 and CD86 expression on C5aR^+^ or C5aR^−^ macrophages in ID8 tumors (n = 4 mice; two-tailed paired t test). (J) Analysis of correlations between *C5aR* expression and M2-like macrophages related markers (*CD209* and *CD163*) based on the RNA-seq results from TCGA database of OCs using TIMER. The corrected partial Spearman’s correlation coefficient and statistical p value are presented. (K) Correlation of *C5aR* expression and *CD8A* based on ESTIMATE algorithm from TCGA database. Data are presented as the mean ± SEM. ∗p < 0.05, ∗∗p < 0.01, ∗∗∗p < 0.001, ∗∗∗∗p < 0.0001; ns, no significance.

Complement is an important component of innate immunity and is overexpressed in various cancer types. Consistent with previous studies, we found a notable increase in C5aR expression in tumor tissues of OC patients relative to adjacent normal tissues (Figures 1A and 1B). C5aR is a receptor for the potent inflammatory mediator C5a and is expressed on various cell types. When determining the cellular origin of C5aR, we did not detect expression of C5aR on the tested human and mouse tumor cell lines (Figure S1A). Instead, we surprisingly found that C5aR exhibited a preferential expression in CD68^+^ macrophages in human OC tumors rather than in CD3^+^ T cells, CD56^+^ NK cells, CD11c^+^ dendritic cells (DCs), or CD66b^+^ neutrophils (Figures 1C–1E). Flow-cytometry analysis of tumors from mice yielded similar results (Figure S1B). Furthermore, it was observed that C5aR was primarily expressed on immunosuppressive CD206^+^ macrophages rather than on CD80^+^ macrophages in human OC tumors (Figure 1F) and mouse tumors (Figure 1G), or on naive or M1-polarized macrophages derived from human peripheral blood mononuclear cells (PBMCs) (Figure 1H) or macrophages from mouse spleen (Figure S1C), suggesting a potential correlation between C5aR expression and macrophage immune function. Consistent with this, C5aR^+^ macrophages infiltrating mouse tumors expressed higher levels of CD206, transforming growth factor β1 (TGF-β1), and IL-4 but much lower levels of CD86 compared to their C5aR^−^ counterparts (Figures 1I and S1D). Moreover, treatment with tumor-cell-derived conditioned medium (TCM) induced high levels of C5aR expression on macrophages derived from PBMCs (Figure 1H), bone marrow-derived macrophages (BMDMs), and THP-1 cells (Figure S1A), suggesting that tumor cells likely have the potential to polarize C5aR-expressing macrophages.

Findings from the Tumor Immune Estimation Resource (TIMER), a comprehensive resource for systematic analysis of immune infiltrates across diverse cancer types,^26^ confirmed that *C5aR* expression was positively correlated with immunosuppressive M2-like macrophages but not correlated with proinflammatory M1-like macrophages (Figure S1E). The expression levels of some well-known immunosuppressive macrophage markers, such as *CD209*, *CD163*, and others,^27^ exhibited a strong positive correlation with the expression of *C5aR* (Figures 1J and S1F). In addition, we detected significantly increased levels of C5aR ligand C5a in the blood of tumor-bearing mice and C5a levels inversely correlated with tumor-infiltrating CD8^+^ T cell ratio (Figures S1G and S1H). Consistently, we also observed a notable inverse association between the expression of *C5aR* and *CD8A* in cancer patients (Figure 1K). Taken together, these results indicated that C5aR expression was upregulated in immunosuppressive TME and was preferentially expressed on TAMs.

### C5aR deficiency restores TAM antitumor activity and slows tumor growth

**Figure 2:**
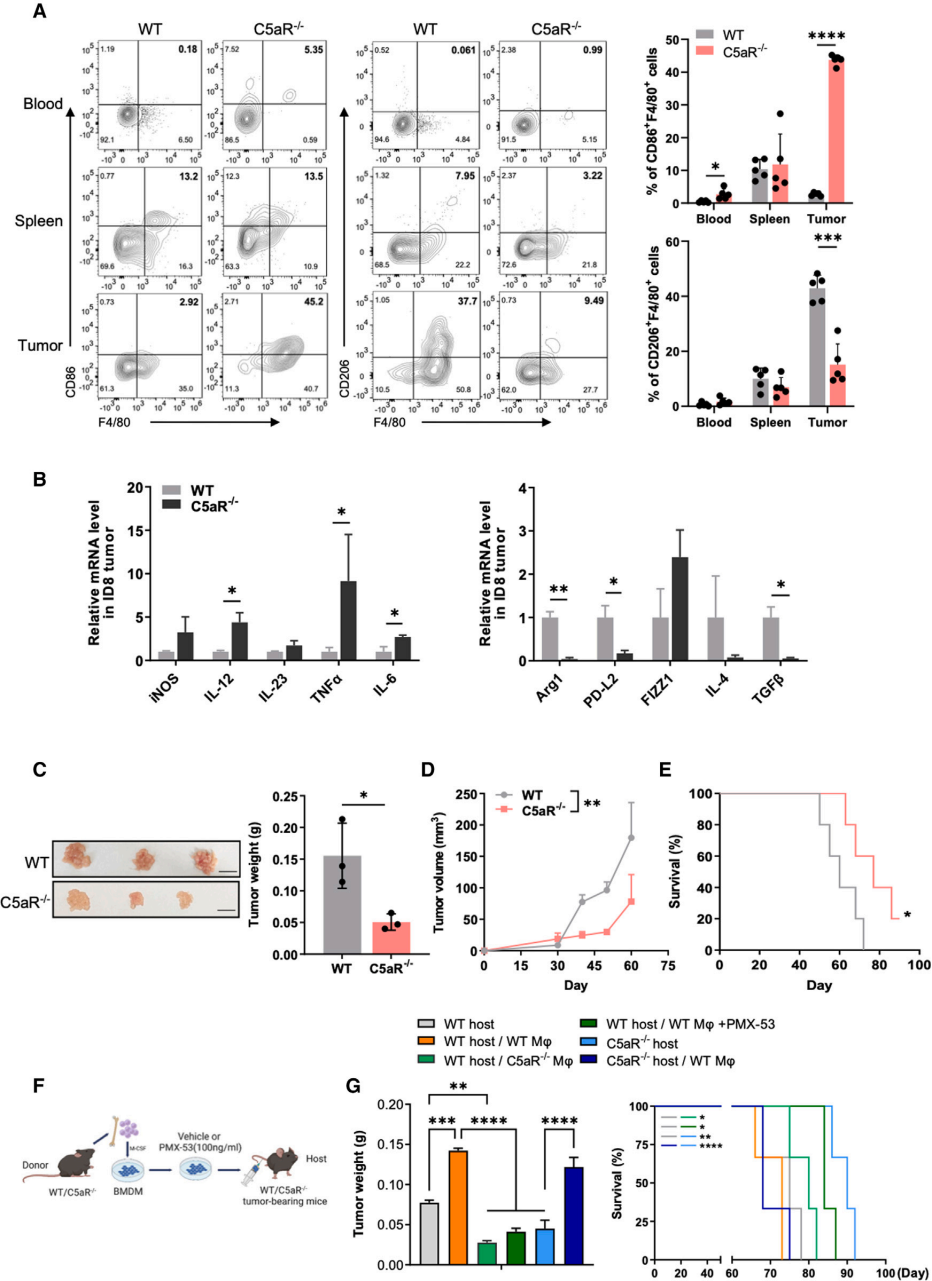
C5aR deficiency restores TAM antitumor activity and slows tumor growth (A) Proportion of peripheral blood, spleen, and tumor-infiltrating CD86^+^F4/80^+^ and CD206^+^F4/80^+^ macrophages measured by flow cytometry in WT and C5aR^−/−^ mice (n = 5 mice; two-way ANOVA). (B) Relative mRNA of M1-like (*iNOS*, *IL-12*, *IL-23*, *TNF-α*, and *IL-6*) and M2-like (*Arg1*, *PD-L2*, *FIZZ1*, *IL-4*, and *TGF-β*) macrophage-related gene expression level in ID8 tumors from WT and C5aR^−/−^ mice (n = 3 independent replicates; two-way ANOVA). (C) Representative images and weight analysis of ID8 tumors from WT and C5aR^−/−^ mice on day 60 (n = 3 mice; two-tailed unpaired t test). Scale bars, 1 cm. (D) ID8 tumor growth in WT and C5aR^−/−^ mice (n = 3 mice at each time point; two-tailed unpaired t test). (E) Survival curve of WT and C5aR^−/−^ ID8 tumor-bearing mice (n = 5 mice; log-rank test). (F) Scheme of establishment of WT and C5aR-deficient BMDM, following C5aR blockade, for sequential adoptive transfer into tumor-bearing mice. (G) Tumor weight (n = 3 mice; one-way ANOVA) and survival curve (n = 3 mice; log-rank test) of tumor-bearing WT and C5aR^−/−^ mice receiving BMDMs (2 × 10^6^ macrophages; intraventricularly) from WT or C5aR^−/−^ mice pretreated with TCM containing C5aR antagonist (PMX-53; 100 ng/mL) or PBS. Results are representative of three independent experiments. Data are presented as the mean ± SEM. ∗p < 0.05, ∗∗p < 0.01, ∗∗∗p < 0.001, ∗∗∗∗p < 0.0001; ns, no significance.

To evaluate the role of C5aR in regulating TAM function during tumor progression, we employed C5aR-deficient (C5aR^−/−^) mice. Tumor cells (ID8) were intraperitoneally injected into wild-type (WT) and C5aR^−/−^ mice. C5aR deficiency did not obviously affect the proliferation of macrophages (Figure S2A). However, genetic blockade or pharmacological inhibition of C5aR significantly improved the cytotoxicity of macrophages against ID8 tumor cells *in vitro* (Figures S2B and S2C). Moreover, we found that CD80 expression was significantly increased and CD163 was attenuated in TAMs from C5aR^−/−^ mice compared to those from WT mice (Figure S2D), suggesting increased TAM activation. Additionally, C5aR-deficient macrophages exhibited a notable decrease in IL-10 expression (Figure S2D), which may be beneficial, as previous reports suggest a detrimental role for macrophage IL-10 in suppressing CD8^+^ T cell-dependent responses to chemotherapy.^28^ CD86 and CD206 expression were utilized to assess macrophage polarization status, which typically indicates whether TAMs have antitumor or protumor activities, although this characterization does not capture the full complexity of TAMs.^29^^,^^30^ Consistent with the above results, C5aR^−/−^ tumor-bearing mice had increased abundance of CD86^+^F4/80^+^ macrophages and decreased frequency of CD206^+^F4/80^+^ macrophages in the TME compared to WT tumor-bearing mice, without significant change in the blood and spleen macrophage population between the two groups (Figure 2A). Similar results were obtained from an immunofluorescence assay, which showed an increase in CD11b^+^F4/80^+^CD80^+^ cells in the tumors of C5aR^−/−^ mice (Figure S3A). Additionally, macrophage effector molecular tumor necrosis factor α (TNF-α) was also upregulated after C5aR blockade (Figure S3B). Quantitative PCR (qPCR) analysis showed that mRNA levels of antitumor molecules, such as *IL-12* and *TNF-α*, were significantly increased while in protumor molecules mRNA levels of arginase-1 (*Arg1*) and *TGF-β* were markedly inhibited in tumors from C5aR^−/−^ mice (Figure 2B), suggesting an immunosuppressive role for C5aR. More importantly, tumor growth and the number of abdominal metastases were markedly decreased in C5aR^−/−^ mice, leading to extended survival compared to WT control mice (Figures 2C–2E and S3C). We further confirmed the improved tumor control resulting from C5aR blockade by introducing luciferase-expressing ID8 cells into both WT and C5aR^−/−^ mice and monitoring tumor size using *in vivo* bioluminescence imaging. The results consistently demonstrated enhanced tumor inhibition in C5aR^−/−^ mice compared to their WT counterparts (Figure S3D).

Since C5aR knockout impacts C5aR activities in all cell types, we next evaluated the contribution of macrophage C5aR in tumor control. We generated BMDMs from C5aR^−/−^ and WT mice, treated with or without PMX-53, a C5aR antagonist,^31^ following their implantation into WT or C5aR^−/−^ tumor-bearing mice (Figures 2F, S3E, and S3F). Mice that received C5aR^−/−^ BMDMs showed reduced tumor burden and improved survival compared to the mice that received WT BMDMs (Figure 2G), which phenocopied the results observed in C5aR^−/−^ tumor-bearing mice. Similarly, transfer of PMX-53-treated WT BMDMs also decreased tumor weight and extended survival in WT recipient mice (Figure 2G). Moreover, implantation of WT BMDMs into either WT or C5aR^−/−^ recipients aggravated tumor growth, leading to a worse survival in these mice (Figure 2G). Together, these data demonstrated that C5aR activation dampens TAM antitumor activity while macrophage C5aR promotes tumor progression. In support of the relevance of this mechanism in human OC, we observed that low C5aR expression and high M1-like macrophage expression significantly prolonged patient survival (Figure S3G).

### Inhibition of C5aR on macrophage augments CD8^+^ T cell antitumor response

**Figure 3:**
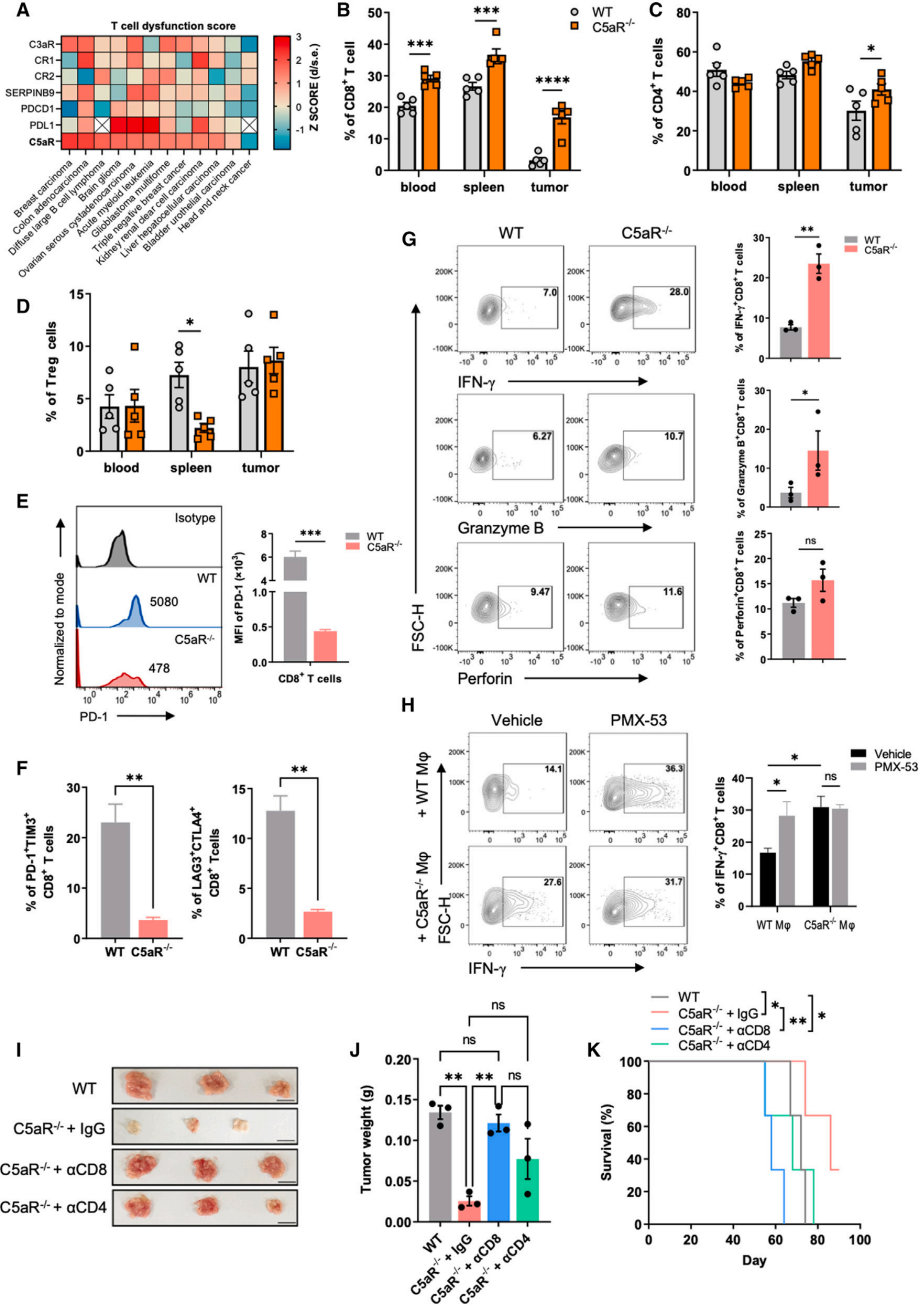
C5aR deficiency alleviates immunosuppression of TME (A) T cell dysfunction scores of C3aR and C5aR in indicated cancer types assessed by Tumor Immune Dysfunction and Exclusion (TIDE) score. T cell dysfunction score is defined as the *Z* score of d/standard error (d/s.e.). (B–D) Proportion of peripheral blood, spleen, and tumor-infiltrating CD8^+^ T cells (B), CD4^+^ T cells (C), and regulatory T cells (Tregs) (D) measured by flow cytometry on day 60 after ID8 tumor inoculation in WT and C5aR^−/−^ mice (n = 5 mice; two-way ANOVA). (E) Flow-cytometry analysis of expression of PD-1 in CD8^+^ T cells from WT and C5aR^−/−^ mice (n = 3 mice; two-tailed unpaired t test). (F) Proportions of PD-1^+^TIM3^+^ and LAG-3^+^CTLA4^+^ cells of tumor-infiltrating CD8^+^ T cells from WT and C5aR^−/−^ mice were measured using flow cytometry (n = 3 mice; two-tailed unpaired t test). (G) Proportions of tumor-infiltrating IFN-γ^+^CD8^+^ T, granzyme B^+^CD8^+^ T, and perforin^+^CD8^+^ T cells from WT and C5aR^−/−^ mice were measured using flow cytometry (n = 3 mice; two-tailed unpaired t test). (H) Proportion of IFN-γ^+^CD8^+^ T cells of murine splenic lymphocytes culture mixed with WT BMDMs or C5aR^−/−^ BMDMs (1:1 ratio) in TCM with or without PMX-53 was measured by flow cytometry (n = 3 independent replicates; two-way ANOVA). (I and J) Representative images (I) and quantification of tumor weight (J) of mice at the endpoint after αCD4 (200 μg; intraperitoneally), αCD8 (200 μg; intraperitoneally), or IgG control therapy (n = 3 mice; one-way ANOVA). Scale bars, 1 cm. (K) Survival curve of tumor-bearing mice receiving antibody treatment (n = 3 mice; log-rank test). Results are representative of three independent experiments. Data are presented as the mean ± SEM. ∗p < 0.05, ∗∗p < 0.01, ∗∗∗p < 0.001, ∗∗∗∗p < 0.0001; ns, no significance.

Next, we sought to find out whether C5aR expression impacts the tumor immune microenvironment. As T cells play a crucial role in limiting tumor growth, we first assessed the correlation between C5aR expression and T cell function in cancer patients. By using the Tumor Immune Dysfunction and Exclusion computational framework (TIDE),^32^ we evaluated the effects of C5aR expression on T cell dysfunction in patient samples obtained from The Cancer Genome Atlas (TCGA). As expected, *C5aR* expression was found to result in high T cell dysfunction scores in various cancer types, surpassing even the well-known T cell exhaustion-inducing markers, programmed cell death 1 (*PDCD1*) and programmed cell death-ligand 1 (*PDL1*) (Figure 3A). In contrast, other complement receptors, such as *CR1*, *CR2*, and *C3aR*, had a comparatively lower effect on T cell dysfunction (Figure 3A). Based on this observation, we hypothesized that inhibition of C5aR could restore T cell antitumor activity. Indeed, our study showed that in C5aR-deficient mice, there was a significant increase in the frequency of CD8^+^ and CD4^+^ T cells in the TME (Figures 3B and 3C), which was confirmed by immunofluorescence assay (Figure S4A). Additionally, we observed an increase in the proliferation of tumor-infiltrating CD8^+^ T cells after C5aR depletion as indicated by 5-ethynyl-2′-deoxyuridine (EdU) expression (Figure S4B). We did not detect any change in the abundance of intratumoral regulatory T cells (Tregs) between WT and C5aR^−/−^ mice (Figure 3D). As PD-1 (encoded by *PDCD1*), cytotoxic T lymphocyte antigen 4 (CTLA4), thymocyte selection-associated high-mobility group box protein (TOX), T cell immunoglobulin and mucin domain-containing protein 3 (TIM3), and signaling lymphocytic activation molecule family member 6 (SLAMF6) are important immune regulators that impact T cell immune responses,^33^ we next examined their expression on cytotoxic T lymphocytes after C5aR blockade. The results showed a significant decrease in the expression of these cell markers in CD8^+^ T cells and a reduced frequency of CD8^+^ T cells that are double-positive for Tim-3 and PD-1, as well as CTLA4 and LAG3, in tumors from C5aR^−/−^ mice compared to WT controls (Figures 3E, 3F, S4C, and S4D), in agreement with gene expression data (Figure S4E). However, higher proportions of interferon-γ^+^ (IFN-γ^+^) and granzyme B^+^ (GzmB) CD8^+^ T cells were observed in the tumors from C5aR^−/−^ mice compared to WT mice (Figure 3G), consistent with results from immunofluorescent staining and qPCR analysis (Figures S5A and S5B), indicating enhanced cytotoxic T lymphocyte responses. Additionally, C5aR deficiency did not induce significant changes in DCs, neutrophils, and NK cells within the TME (Figure S5C).

To assess whether macrophage C5aR directly regulates T cell activation, WT or C5aR^−/−^ macrophages were co-cultured with splenic T cells in the presence or absence of PMX-53. We found that C5aR deletion abolished macrophage-mediated T cell suppression, characterized by increased expression of cytotoxic and cytolytic effector molecules in T cells (Figures 3H and S5D). Importantly, PMX-53 treatment of C5aR^−/−^ macrophages had no additional effect on T cell activation compared with vehicle-treated C5aR^−/−^ macrophages (Figures 3H and S5D), indicating that the effect of the drug on macrophages is mediated by C5aR. Additionally, results from macrophage adoptive transfer experiments demonstrated that implantation of C5aR^−/−^ BMDMs or PMX-53-treated WT BMDMs markedly enhanced the activity of tumor-infiltrating CD8^+^ T cells, while transfer of WT BMDMs significantly suppressed CD8^+^ T cell activity (Figures S5E and S5F). To further verify that macrophage C5aR controls T cell antitumor response, splenic T cells were co-cultured with WT BMDMs or C5aR^−/−^ BMDMs in the presence or absence of PMX-53 and then implanted into recipient mice. Tumor growth was significantly inhibited in the host receiving T cells educated by C5aR^−/−^ or PMX-53-treated WT BMDMs compared to those receiving WT BMDM-treated T cells (Figure S5G). To determine whether C5aR deficiency conferred better tumor control depending on enhanced T cell responses, we depleted CD8^+^ T cells or CD4^+^ T cells in C5aR^−/−^ tumor-bearing mice with anti-CD8 or anti-CD4 antibodies. CD8^+^ T cell depletion completely abrogated the inhibition of tumor growth in C5aR^−/−^ mice, whereas CD4^+^ T cells had a partial effect (Figures 3I–3K), indicating that the enhanced antitumor immunity depends on CD8^+^ T cells. Together, these data indicated that C5aR activation on macrophage inhibits CD8^+^ T cell antitumor activity and promotes tumor progression.

### C5aR activity shapes the transcriptional landscape of macrophages

**Figure 4:**
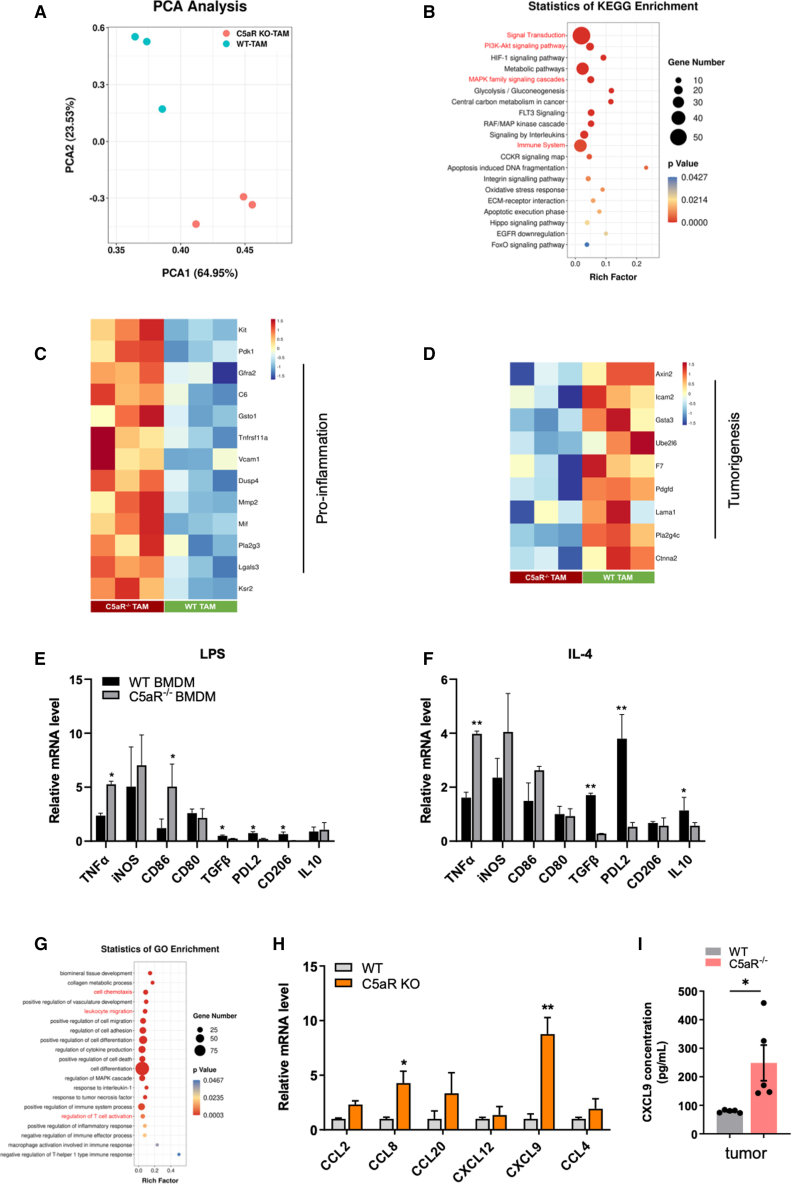
C5aR activity shapes the transcriptional landscape of macrophages (A) Principal component analysis (PCA) of RNA-seq samples extracted from WT or C5aR^−/−^ TAM *in vivo* (n = 4 mice per group). (B) KEGG enrichment analysis of upregulated differentially expressed genes between WT or C5aR^−/−^ TAM. (C and D) Heatmap depicting relative expression of proinflammation (C) or tumorigenesis (D) related genes in differentially expressed genes between WT or C5aR^−/−^ TAM. (E and F) Relative mRNA expression of *TNF-α*, *iNOS*, *CD86*, *CD80*, *TGF-β*, *PD-L2*, *CD206*, and *IL-10* in WT or C5aR^−/−^ BMDM stimulated by 50 ng/mL LPS (E) or 40 ng/mL IL-4 (F) (n = 3 independent replicates; two-way ANOVA). (G) Gene ontology (GO) enrichment analysis of differentially expressed genes between WT or C5aR^−/−^ TAM. (H) Relative mRNA of chemokine genes (*CCL2*, *CCL8*, *CCL20*, *CXCL12*, *CXCL9*, and *CCL4*) expression level in ID8 tumors from WT and C5aR^−/−^ mice (n = 3 independent replicates; two-way ANOVA). (I) Supernatants of ID8 tumors from WT or C5aR^−/−^ mice were harvested for quantification of CXCL9 production by ELISA assay (n = 5 mice; two-tailed unpaired t test). Data are presented as the mean ± SEM. ∗p < 0.05, ∗∗p < 0.01, ∗∗∗p < 0.001, ∗∗∗∗p < 0.0001; ns, no significance.

To further address the mechanisms of C5aR-mediated protumoral phenotype of TAMs, we conducted *in vivo* sorting of TAMs from both WT and C5aR^−/−^ mice followed by RNA sequencing (RNA-seq) analysis. Principal component and hierarchical cluster analysis revealed that C5aR^−/−^ macrophages were transcriptionally distinct from WT macrophages (Figures 4A and S6A). Pathways related to macrophage antitumor function such as signal transduction, T cell activation, and MAPK (mitogen-activated protein kinase) signaling were significantly upregulated in C5aR^−/−^ macrophages (Figures 4B and S6B), concomitant with downregulated immunosuppressive IL-4 and IL-13 signaling pathway (Figure S6C). We also observed upregulation of various proinflammatory cytokine and innate immunity genes in C5aR^−/−^ macrophages compared with WT macrophages (Figures 4C and S6D), whereas genes associated with tumorigenesis and anti-inflammation were inhibited (Figures 4D and S6E). These results are consistent with the previously mentioned data that showed an increase in macrophage activity following C5aR deletion (Figure 2), again implying that C5aR controls macrophage antitumor response.

To confirm the results obtained from RNA-seq analysis, we investigated the activity of primary murine macrophages derived from either WT or C5aR^−/−^ mice, which were stimulated *in vitro* with lipopolysaccharide (LPS) or IL-4 under macrophage colony-stimulating factor (M-CSF) conditions. The results showed that genes associated with proinflammatory and M1 polarization were upregulated in C5aR^−/−^ macrophages, while genes associated with anti-inflammatory and M2 polarization were inhibited compared to WT macrophages (Figures 4E and 4F). Consistent with this, an increased abundance of M1-like cells was observed in macrophages from C5aR^−/−^ mice under proinflammatory stimulation, whereas a decrease in M2-like macrophages was observed under anti-inflammatory stimulation (Figure S6F). Additionally, C5aR^−/−^ macrophages showed a significant increase in M1 polarization and a tendency toward decreased M2 polarization under TCM conditions (Figure S6G). Together, these data suggest that C5aR is a key driver of TAM phenotype, and loss of C5aR contributes toward TAM switching from a protumor phenotype to an antitumor phenotype with heightened proinflammatory responses.

In addition, an intriguing result emerged from our analysis of the transcriptional data, indicating that the loss of C5aR led to an upregulation of cell chemotaxis and leukocyte migration response in macrophages (Figure 4G). Since chemokine-mediated recruitment of T cells plays an important role in antitumor immunity, we then asked whether C5aR impacts macrophage chemokine expression. As expected, increased expression of chemokine (C-C motif) ligand 8 (*CCL8*), chemokine (C-C motif) ligand 2 (*CCL2*), and chemokine (C-X-C motif) ligand 9 (*CXCL9*) were found in C5aR^−/−^ macrophages, and *CXCL9* was the one most affected by C5aR (Figure 4H). In agreement with this, we detected markedly increased protein levels of CXCL9 in the tumors and blood of C5aR^−/−^ mice compared with those of WT controls (Figures 4I and S6H).

### C5aR deficiency enhances TAM-mediated CXCL9 secretion and recruitment of CD8^+^ T cells

**Figure 5:**
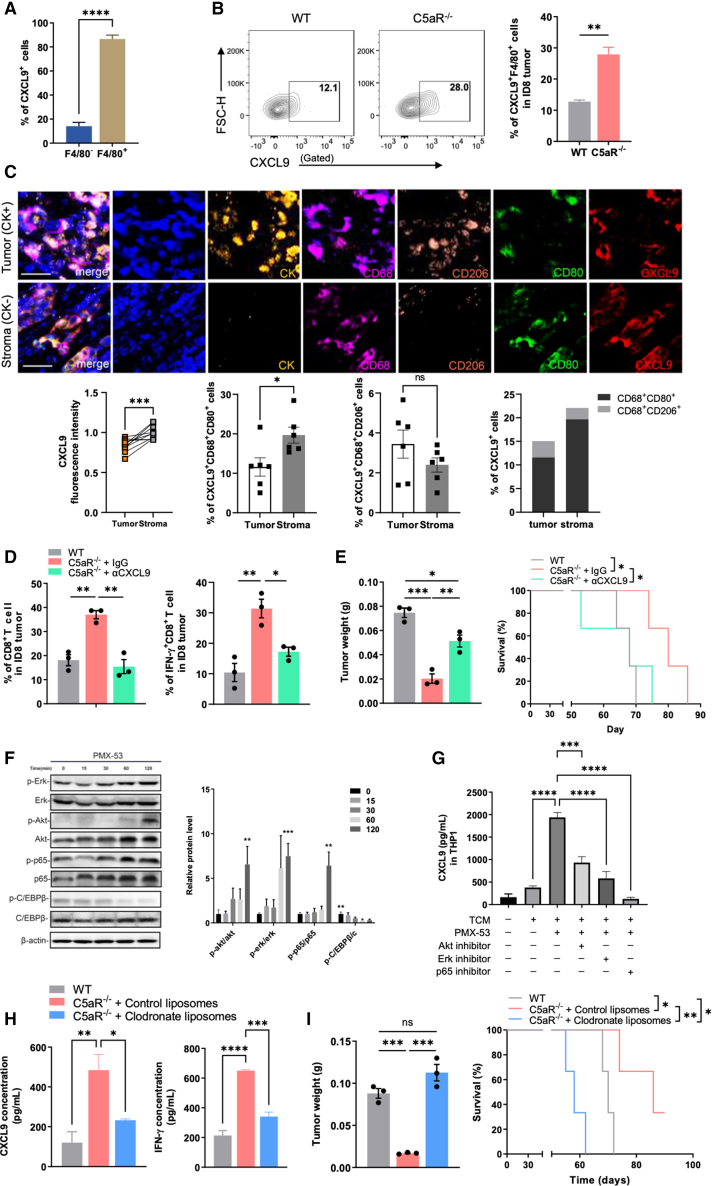
C5aR deficiency enhances TAM-mediated CXCL9 secretion and recruitment of CD8^+^ T cells (A) Proportions of F4/80^−^ and F4/80^+^ cells among CXCL9^+^ cells in ID8 tumors from WT mice were measured using flow cytometry (n = 3 mice; two-tailed paired t test). (B) Proportions of tumor-infiltrating CXCL9^+^F4/80^+^ cells were measured using flow cytometry from WT and C5aR^−/−^ mice (n = 3 mice; two-tailed unpaired t test). (C). Representative images and quantification of CXCL9 fluorescence intensity, and proportion of CXCL9^+^CD68^+^CD80^+^ and CXCL9^+^CD68^+^CD206^+^ cells in CK^+^ or CK^−^ region of human OC tissues (n = 6 samples; two-tailed paired t test). Scale bars, 25 μm. (D) Proportion of tumor-infiltrating CD8^+^ T and IFN-γ^+^CD8^+^ T cells from WT and C5aR^−/−^ mice receiving αCXCL9 (200 μg; intraperitoneally) or IgG treatment was measured by flow cytometry (n = 3 mice; one-way ANOVA). (E) Tumor weight (n = 3 mice; one-way ANOVA) and survival curve (n = 3 mice; log-rank test) of tumor-bearing mice receiving αCXCL9 or IgG treatment. (F) Representative western blot gel documents and summarized data showing the activity of ERK/AKT/NF-κB p65 and C/EBPβ in TCM-educated THP1-derived macrophages stimulated with PMX-53 (100 ng/mL). β-Actin was used as a loading control (n = 3 independent replicates; two-way ANOVA). (G) Supernatants of THP1 cells were harvested for quantifying CXCL9 production by ELISA assay (n = 3 independent replicates; one-way ANOVA). (H) Serum of ID8 tumor-bearing WT or C5aR^−/−^ mice receiving clodronate liposomes (200 μL; intraperitoneally) or control liposomes (200 μL; intraperitoneally) treatment were harvested for quantifying CXCL9 and IFN-γ production by ELISA assay (n = 3 mice; one-way ANOVA). (I) Tumor weight (n = 3 mice; one-way ANOVA) and survival curve (n = 3 mice; log-rank test) of tumor-bearing mice receiving clodronate liposomes or control liposomes treatment. Results are representative of three independent experiments. Data are presented as the mean ± SEM. ∗p < 0.05, ∗∗p < 0.01, ∗∗∗p < 0.001, ∗∗∗∗p < 0.0001; ns, no significance.

To confirm the contribution of macrophage C5aR blockade to increased CXCL9 expression, we performed flow-cytometry analysis of TAMs from tumors in both WT and C5aR^−/−^ mice. We found that the majority of CXCL9^+^ cells were F4/80 positive, and loss of C5aR significantly increased the abundance of CXCL9^+^F4/80^+^ macrophages in tumors (Figures 5A and 5B). Notably, immunofluorescence analysis of tumors from OC patients revealed significantly lower levels of CXCL9 in tumor tissues compared to adjacent normal regions (Figure 5C). CXCL9 predominantly exhibited expression within macrophages that are positive for CD68 and CD80, as opposed to CD68^+^CD206^+^ macrophages (Figure 5C), corresponding to a result obtained from Gene Expression Profiling Interactive Analysis (GEPIA)^34^ of patient samples from TCGA databases, which showed a tight correlation between M1-like macrophages and CD8^+^ T cells (Figure S7A). Additionally, PMX-53 treatment significantly upregulated the protein levels of CXCL9 and TNF-α in the medium of THP-1 cells compared to vehicle-treated control (Figures 5G and S7F). These results suggest that C5aR activation on macrophages suppresses CXCL9 expression.

Since CXCL9 has been reported to attract immune cells,^35^ we next determined whether CXCL9 mediates recruitment of CD8^+^ T cells *in vitro* and *in vivo*. Transwell assay showed that C5aR^−/−^ BMDMs promoted migration of CD8^+^ T cells as compared to WT BMDMs (Figure S7B). However, addition of anti-CXCL9 antibodies returned the C5aR^−/−^ BMDM-mediated accumulation of CD8^+^ T cells to the levels of the WT control (Figure S7B). We next depleted CXCL9 in WT and C5aR^−/−^ tumor-bearing mice. Decreased frequency of CD8^+^ T cells was observed in tumors from C5aR^−/−^ mice that received anti-CXCL9 antibodies, and the expression of IFN-γ, GzmB, and perforin in CD8^+^ T cells was restored to the levels of WT controls (Figures 5D and S7C). Notably, the inhibition of tumor growth and improved survival in C5aR^−/−^ mice was abrogated by depleting CXCL9 (Figure 5E), suggesting that the enhanced antitumor function by C5aR deletion depends on CXCL9-mediated recruitment of CD8^+^ T cells. To support this mechanism in human OC, we observed a significant correlation between *CXCL9* and the expression of *CD8A*, *IFNG*, perforin 1 (*PRF1*), granzyme A (*GZMA*), *GZMB*, and chemokine (C-X-C motif) receptor 3 (*CXCR3*) (Figure S7D).

To understand how C5aR regulates macrophage CXCL9 secretion, we conducted western blotting analysis to identify the intracellular pathway affected by C5aR signaling. Since nuclear factor κB (NF-κB) has been previously reported to enhance the expression of proinflammatory cytokines,^36^ and CCAAT/enhancer binding protein β (C/EBPβ) can stimulate the expression of immunosuppressive molecules,^37^ we investigated the activation of NF-κB/p65 and C/EBPβ. We found that PMX-53 treatment upregulated phosphorylation p65 while it suppressed phosphorylation of C/EBPβ (Figures 5F and S7E). We next examined the impact of C5aR inhibition on the phosphorylation of proteins associated with NF-κB activation, including extracellular regulated protein kinases and protein kinase B. Our findings revealed that treating TCM-educated THP-1 cells with PMX-53 resulted in increased phosphorylation of both ERK and AKT (Figures 5F and S7E). To investigate whether the ERK/AKT/NF-κB axis is responsible for the increased expression of CXCL9 observed after blocking C5aR, THP-1 cells were treated with PMX-53 and inhibitors targeting ERK, AKT, or NF-κB/p65, and results showed that the upregulation of CXCL9 expression in THP-1 cells was reversed by the addition of an inhibitor specific to ERK, AKT, or NF-κB (Figures 5G and S7F). We also observed increased activation of signal transducer and activator of transcription 1 (STAT1) and suppression of STAT6 in response to PMX-53 treatment (Figure S7G), two key transcription factors involved in macrophage polarization, echoing the results that C5aR expression modulates macrophage transcription. Therefore, these results indicate that the activation of C/EBPβ and inhibition of NF-κB signaling by C5aR are responsible for the suppression of macrophage CXCL9 expression.

To further verify that macrophage C5aR controls CXCL9 expression and tumor progression, we treated WT and C5aR^−/−^ tumor-bearing mice with clodronate liposomes, which have been widely used to deplete macrophages *in vivo*.^38^ After the deletion of macrophages, the protein level of CXCL9 and IFN-γ in the serum of C5aR^−/−^ mice decreased significantly and was similar to that of the WT control (Figure 5H). Simultaneously, the protein levels of TNF-α also markedly decreased in C5aR^−/−^ mice (Figure S7H). In agreement with this, flow-cytometry analysis revealed that the frequency of intratumoral CD8^+^ T cells and the CD8^+^ T cells expressing IFN-γ in C5aR^−/−^ mice returned to the levels of WT tumor-bearing mice, along with increased PD-1 expression after macrophage depletion, leading to increased tumor weight and worsening survival in C5aR^−/−^ mice (Figures 5I and S7I). Importantly, clodronate liposome treatment effectively inhibited tumor growth in WT tumor-bearing mice, but the combination with C5aR inhibitor had no additional effects, indicating that C5aR in macrophages, and not other cell types, promotes tumor growth (Figure S7J). These data suggest that C5aR regulates CXCL9 expression by macrophages and corroborate the notion that macrophage C5aR blockade enhances T lymphocyte antitumor immunity.

### C5aR expression correlates with cancer patient outcomes and abundance of CXCL9 and CD8^+^ T cells

**Figure 6:**
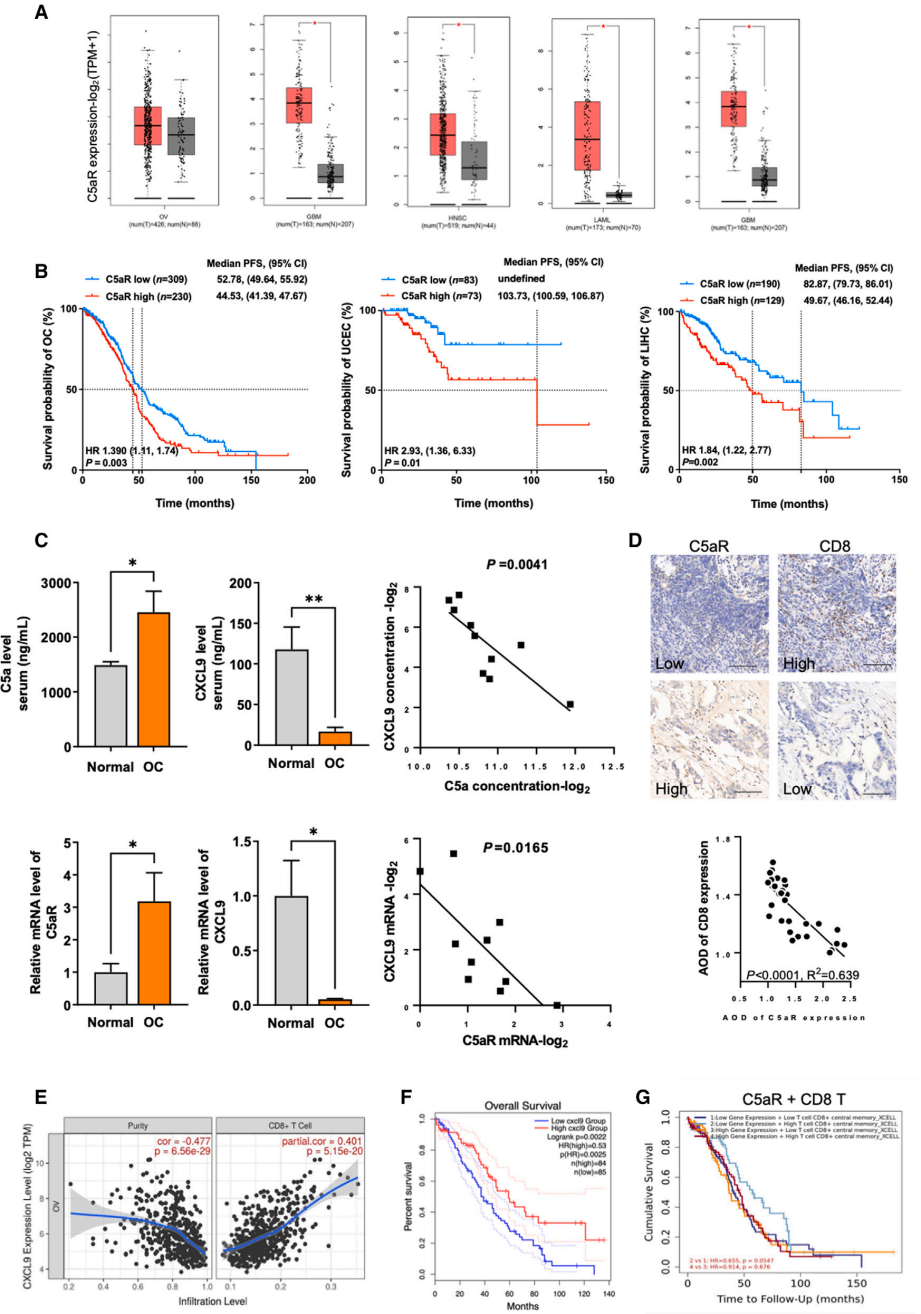
Aberrant expression of C5aR correlates with low abundance of CXCL9 and CD8^+^ T cells and poor outcome in cancer patients (A) *C5aR* expression in multiple types of tumor samples and corresponding normal samples from RNA-seq results of TCGA database using GEPIA website. OV, ovarian serous cystadenocarcinoma; GBM, glioblastoma multiforme; HNSC, head and neck squamous cell carcinoma; LAML, acute myeloid leukemia; ESCA, esophageal carcinoma. Data were analyzed by two-tailed unpaired t test. (B) Kaplan-Meier analysis of the relationship between *C5aR* mRNA expression and prognosis of various tumors from TCGA dataset. C5aR low: patients with a low mRNA expression of *C5aR*; C5aR high: patients with a high mRNA expression of *C5aR*. OV, ovarian serous cystadenocarcinoma; UCEC, uterine corpus endometrial carcinoma; LIHC, liver hepatocellular carcinoma. Data were analyzed by log-rank test with p value showing statistical difference. Median overall survival (OS) and hazard ratio (HR) are shown. (C) Serum concentrations of C5a and CXCL9 in OC and normal patients and correlation analysis between them (upper panels). Expression levels of *C5aR* and *CXCL9* mRNA in ovarian tissues of OC and normal patients and correlation analysis between them (lower panels). n = 5 samples; two-tailed unpaired t test. R^2^ and p values of Pearson correlation analysis. (D) Representative immunohistochemistry images of OC tumor tissues with low or high C5aR expression and corresponding CD8 expression in the same cases (n = 15 samples). Scale bars, 100 μm. R^2^ and p values of Pearson correlation analysis between the AOD value of C5aR and CD8 are presented. (E) Analysis of correlations between *CXCL9* expression and tumor purity and CD8^+^ T cells in TCGA basal-like OC using TIMER. The purity-corrected partial Spearman’s correlation coefficient and statistical p value are presented. (F) Kaplan-Meier analysis of the relationship between the *CXCL9* mRNA expression and prognosis of ovarian cancer from TCGA dataset (log-rank test). p value shows the statistical difference. (G) Kaplan-Meier analysis of the relationship between the *C5aR* mRNA expression level in synergy with the degree of CD8^+^T cell tumor infiltration and prognosis of OC patients from TCGA dataset. p value shows the statistical difference. Data are presented as the mean ± SEM. ∗p < 0.05, ∗∗p < 0.01, ∗∗∗p < 0.001, ∗∗∗∗p < 0.0001; ns, no significance.

The findings reported above indicated that C5aR modulates TAM antitumor activities, thereby affecting the recruitment and function of CD8^+^ T cells. To validate these results in cancer patients, we first examined the prognostic value of C5aR. We found that multiple types of cancer exhibited upregulated *C5aR* expression, and high *C5aR* expression significantly correlated with poor survival in various types of cancer (Figures 6A, 6B, S8A, and S8B), in agreement with previous reports showing elevated C5a levels linked with poor prognosis in cancer patients.^11^^,^^39^ Additionally, we observed strong and positive correlation of the *C5aR* with T cell exhaustion signature genes in OC patients based on TCGA database of RNA-seq data, including lymphocyte activating 3 (*LAG3*), *PDCD1*, *CTLA4*, and T cell immunoreceptor with immunoglobulin and immunoreceptor tyrosine-based inhibition motif domains (*TIGIT*)^33^ (Figure S8C), echoing our findings that C5aR deficiency decreased immune checkpoint molecular expression on cytotoxic T lymphocytes (Figures 3E and S4C). More importantly, we collected sera from both healthy women and OC patients and subsequently measured the levels of C5a and CXCL9 using ELISA. Additionally, we obtained normal ovarian tissues confirmed through pathological examination and tissues confirmed to be OC. We then assessed the protein and mRNA expression levels of C5aR and CXCL9. We observed elevated levels of C5a and C5aR alongside decreased CXCL9 levels in the serum and tissues of OC patients compared to healthy controls (Figures 6C and S8D). Moreover, we identified a negative correlation between C5aR expression and CXCL9 levels (Figures 6C and S8D). Consistent with this, we observed that C5aR plays a role in determining the abundance of CD8^+^ T cells in tumor tissues from patients with OC (Figure 6D). Accordingly, *CXCL9* strongly correlated with CD8^+^ T cell abundance in tumors, and high *CXCL9* expression predicted better survival in ovarian and other cancer patients when compared to low *CXCL9* expression (Figures 6E, 6F, and S8E). Patients with low *C5aR* expression and high CD8^+^ T cells exhibited better survival than those with high *C5aR* and low CD8^+^ T cells (Figure 6G). Altogether, the data suggest that low C5aR is beneficial for the upregulation of CXCL9 and infiltration of CD8^+^ T cells in the TME, thus leading to a better survival in cancer patients.

### C5aR inhibition synergizes with PD-1 blockade immunotherapy

**Figure 7:**
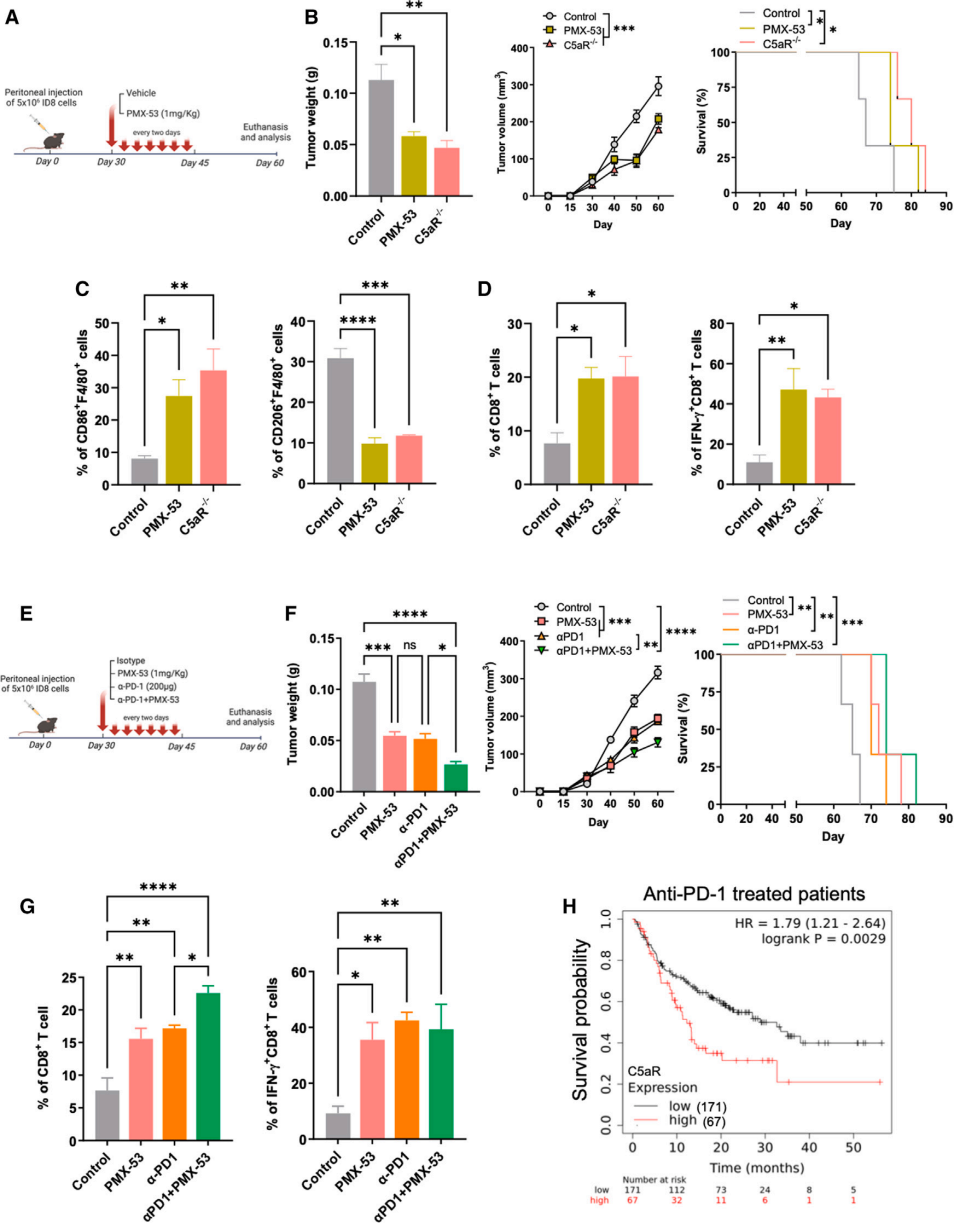
C5aR inhibition synergizes with PD-1 blockade immunotherapy (A) WT tumor-bearing mice were treated with PMX-53 (1 mg/kg; intraperitoneally) or vehicle (PBS) on day 30 and every 2 days thereafter for a total of seven injections, and C5aR^−/−^ tumor-bearing mice served as controls. (B) Tumor weight (n = 3 mice; one-way ANOVA), tumor volume (n = 3 mice; one-way ANOVA), and survival curve (n = 3 mice; log-rank test) of mice. (C and D) Proportion of tumor-infiltrating CD86^+^F4/80^+^ (C, left), CD206^+^F4/80^+^ (C, right), CD8^+^ T (D, left), and IFN-γ^+^CD8^+^ T cells (D, right) was measured by flow cytometry (n = 3 mice; one-way ANOVA). (E) WT tumor-bearing mice were injected with αPD-1 (200 μg; intraperitoneally) or PMX-53 (1 mg/kg; intraperitoneally) alone or in combination at day 30, and every 2 days thereafter for a total of seven injections. (F) Tumor weight (n = 3 mice; one-way ANOVA), tumor volume (n = 3 mice; one-way ANOVA), and survival curve (n = 3 mice; log-rank test) of mice. (G) Proportion of tumor-infiltrating CD8^+^ T cells and IFN-γ^+^CD8^+^ T cells from WT mice receiving treatment of αPD-1 or PMX-53 alone or in combination was measured by flow cytometry (n = 3 mice; one-way ANOVA). (H) Comparison of overall survival in the combined three cancer types (bladder, glioblastoma, melanoma ovarian) patients who had high *C5aR* versus low *C5aR* intratumoral expression receiving anti-PD-1 (pembrolizumab) treatment (log-rank test). p value shows the statistical difference. Results are representative of three independent experiments. Data are presented as the mean ± SEM. ∗p < 0.05, ∗∗p < 0.01, ∗∗∗p < 0.001, ∗∗∗∗p < 0.0001; ns, no significance.

Considering the crucial role of C5aR in controlling immune infiltrate phenotype and tumor growth, we next investigated whether pharmacologic inhibition of C5aR has potential value in OC therapy. Mice were intraperitoneally injected with ID8 tumor cells, followed by PMX-53 treatment by day 30 post tumor inoculation when tumor size reached 10 mm^3^ (Figure 7A). Results showed that C5aR inhibition also efficiently reduced tumor weight and extended survival of mice after tumor was established (Figure 7B). PMX-53 did not impede tumor progression in the absence of C5aR (Figure S9A). To investigate the possible mechanism driving the therapeutic effect of PMX-53 treatment, we examined the phenotype of TAMs and the infiltration of CD8^+^ T cells in tumors from mice treated with PMX-53 treatment or vehicle control. More CD86^+^F4/80^+^ macrophages and an increased proportion of CD8^+^ T cells and IFN-γ^+^CD8^+^ T cells were present in mice receiving PMX-53 treatment compared to vehicle-treated controls (Figures 7C and 7D). Similarly, inhibiting C5aR increased CXCL9 expression in macrophages (Figure S9B). To test whether C5aR inhibition potentiates immune checkpoint blockade (ICB) therapy, mice bearing tumors were treated with anti-PD-1 alone or combined with PMX-53 (Figure 7E). Anti-PD-1 treatment also increased CXCL9 expression in TAM (Figure S9C), consistent with previous reports revealing that CXCL9 is required for efficacious ICB therapy.^40^ Moreover, combination treatment exhibited a better therapeutic effect than PD-1 blockade alone, as signified by enhanced antitumor TAMs, higher proportion of macrophages expressing CXCL9, increased frequency of CD8^+^ T cells, and decreased tumor burden (Figures 7F, 7G, S9C, and S9D), suggesting a potential for the combination of PMX-53 and anti-PD-1 in OC immunotherapy. We did not observe significant changes in DCs, neutrophils, or NK cells in response to single or combination treatments (Figure S9E). To bolster the practicality of this finding, we noticed that patients who received anti-PD-1 therapy experienced improved overall survival with low *C5aR* expression as compared to patients with high *C5aR* expression (Figure 7H). Hence, our data suggest that either C5aR knockout or pharmacologic inhibition efficiently slowed tumor progression, again implying a key role for C5aR in controlling the immune state in TME by modulating TAM phenotype and cytotoxic T cell function.

## Discussion

While previous research has explored the roles of complement-derived mediators and macrophages in tumor growth and metastasis in other cancer types,^41^^,^^42^ their functional interplay within the context of OC has remained unexplored. Despite earlier studies indicating the necessity of macrophage-derived CXCL9 for CD8^+^ T cell infiltration,^35^ the key factors governing CXCL9 expression in macrophages have remained elusive. Our data unveil a C5aR-driven mechanism that hinders the production of CXCL9 by macrophages. These findings underscore the intricate connections between C5aR, macrophages, and CXCL9 production within the realm of cancer immunity. Additionally, our study reveals a novel mechanism wherein C5a inhibits CD8^+^ T cell antitumor responses by mediating macrophage C5aR, which contrasts with previous research indicating the direct role of C5a in regulating CD8^+^ T cell function.^14^ Unlike prior studies demonstrating C5aR’s role in enhancing the inhibitory properties of MDSCs to support tumor progression,^43^ our study reveals a distinct protumoral mechanism involving macrophage C5aR. Additionally, our findings indicate a positive correlation between C5aR expression and T cell dysfunction. Moreover, the influence of C5aR expression on the survival of patients undergoing anti-PD-1 therapy suggests its potential as a valuable biomarker for evaluating T cell dysfunction and predicting the potential efficacy of ICB therapy on an individual patient basis.

C5aR is a G-protein-coupled receptor that is expressed on the surface of many cell types, including tumor cells, endothelial cells, lymphocytes, and myeloid cells.^44^^,^^45^^,^^46^ Some tumor cells have been shown to express C5aR, and this expression has been linked to tumor progression.^5^^,^^47^^,^^48^ In our study, we found that C5aR expression was not detected in both human and mouse OC cell lines, indicating that the mechanisms of complement receptor expression and effector pathways that promote tumor progression may differ among different cancer models and across various organs. Consistent with a prior study demonstrating the absence of C5aR expression on CD4^+^ and CD8^+^ T cells,^12^^,^^23^ we similarly did not observe C5aR expression on non-immune cells and lymphocytes. Instead, our observation from OC patients and experimental mice demonstrated that C5aR was mainly expressed on the TAMs, especially those exhibiting a protumoral phenotype. Further study suggests that aberrant C5aR expression suppresses TAM antitumor activity, as C5aR knockout or pharmacologic inhibition enhanced the ability of macrophages to kill tumor cells *in vitro* and increased the abundance of M1-like TAMs *in vivo*, in agreement with previous reports showing the role of C5a-C5aR in regulating the phenotype of macrophages in colon cancer.^25^ Given that C5aR is also expressed in other cells, we performed macrophage co-implantation experiments, the results of which indicated that loss of C5aR on TAMs is the major contributor to the enhanced immunity observed in C5aR^−/−^ mice. Upon binding to C5a, C5aR activates intracellular signaling pathways that lead to changes in gene expression and altered cellular functions. However, the exact mechanism by which C5a-C5aR signaling regulates macrophage transcription is still not determined. We therefore conducted a transcriptome analysis of macrophages treated with TCM in both WT and C5aR-deficient mice. Our results indicate that C5aR expression is a reliable indicator of macrophage suppression, and the two cell populations can be primarily differentiated based on their C5aR expression. Furthermore, C5aR activity appears to inhibit immune-stimulating gene sets and promote immune-suppressing gene modules. Based on these data, we conclude that C5aR is a molecular switch that controls the immune status of macrophages in TMEs.

CD8^+^ T cells are key drivers of antitumor immunity, and extensive evidence has indicated a correlation between infiltration, activation of CD8^+^ T cells, and prognosis in many tumor types.^49^^,^^50^ Chemokines are a class of small cytokines that are secreted by cells and regulate immune cell trafficking and recruitment into tissues, including tumors.^51^^,^^52^ Research has demonstrated that CXCL9 plays a role in attracting immune cells, such as CD8^+^ T cells, to the site of the tumor.^35^^,^^53^ Other studies also showed that the presence of CXCL9 and CXCL10 is necessary for a successful immune response against tumors after ICB.^40^^,^^54^ Consistent with these findings, we found that CXCL9 is essential for antitumor immunity, as CXCL9 neutralization blocked the infiltration of CD8^+^ T cells *in vitro* and *in vivo*. Intriguingly, we observed that depletion of CXCL9 also led to a significant reduction in the abundance of tumor-associated macrophages (data not shown). Since monocytes themselves express CXCR3, the receptor for CXCL9, it is possible that the increased CXCL9 production leads to the recruitment of more macrophages in a positive feedback loop when C5aR is absent. Additionally, it has been reported that macrophages are capable of producing CXCL9 and CXCL10.^40^ Nevertheless, the factors that govern CXCL9 production by macrophages remain elusive. Here we identified a complement component C5aR-driven suppressing mechanism for the macrophage-derived CXCL9. We observed that C5aR deficiency markedly increased protein levels of CXCL9 in the tumors and blood of mice. CXCL9 depletion abrogated the increased proportion of CD8^+^ T cells in the TME and improved survival observed in C5aR^−/−^ mice, indicating the importance of C5aR in controlling CXCL9 production in antitumor immunity. Notably, our *in vitro* experiments demonstrated that although tumor cells could prime macrophages to secrete CXCL9, blockade of C5aR substantially increased CXCL9 production by THP-1 cells, suggesting the potential relevance of C5aR in human cancers. Mechanistically, pharmacologic inhibition of C5aR significantly stimulated phosphorylation of ERK, AKT, and NF-κB/p65 while it suppressed phosphorylation of C/EBPβ, leading to increased production of CXCL9. Blocking ERK, AKT, or NF-κB/p65 with inhibitors reversed C5aR-inhibition-mediated upregulation of the CXCL9 level. Thus, our results indicate that C5a-C5aR signaling controls CXCL9 secretion by macrophages by regulating ERK/AKT/NF-κB pathway activation.

Another important finding of the present study is that C5aR-deficiency-mediated macrophage activation promotes the antitumor function of CD8^+^ T cells. Activation of CD8^+^ T cells within a tumor is a crucial factor that impacts tumor progression.^50^ In the context of cancer, elevated levels of C5a have been detected in the serum of some cancer patients, and these high levels have been associated with a poor prognosis.^11^^,^^39^ A previous report has shown that C5a may directly affect CD8^+^ T cell function by inhibiting the production of IL-10 in an autocrine manner. In the present study, we observed a significant increase in C5a levels after tumor challenge, while C5aR expression was barely detected on T cells, in agreement with previous reports.^12^ When the C5aR was deleted, intratumoral CD8^+^ T cells displayed an improved antitumor response, indicated by increased production of toxic substances as well as reduced expression of exhaustion markers. Furthermore, *in vitro* experiments revealed that co-culture of macrophages with CD8^+^ T cells recapitulated the results observed in C5aR^−/−^ mice, as macrophages were able to directly boost CD8^+^ T cell activation in the absence of C5aR. These results suggest that in addition to its direct effects on CD8^+^ T cells, C5a may also indirectly curb CD8^+^ T cell antitumor activity through macrophage C5aR-mediated inhibition. It is worth noting that the complement system also could impede the antitumor immune response by attracting MDSCs and suppressing NK cell activation in various tumor models. Thus, our study, along with others, shows that the inhibition of host antitumor immunity by complement signaling is complex and multifaceted.

Our results have also established a logical connection between two significant observations from experimental and clinical studies. The first pertains to the impact of C5aR on the antitumor immune response induced by ICB therapy. Results showed that blockade of C5a-C5aR signaling enhanced the antitumor efficacy of PD-1/PD-L1 antibodies.^43^ The second relates to the importance of CXCL9 in the host’s antitumor immunity. As discussed above, experimental and clinical findings indicate that macrophage-derived CXCL9 is required for the infiltration of CD8^+^ T cells and efficacy of ICB therapy.^40^ Our data suggest that C5aR suppresses CXCL9 secretion from macrophages, thereby hindering the recruitment of CD8^+^ T cells, thus impeding host antitumor immunity and promoting tumor progression. Our findings highlight the link between C5aR, macrophages, and CXCL9 production in cancer immunotherapy. Our clinical investigation further substantiated the mechanistic link between C5aR, CXCL9, and CD8^+^ T cells. We found that high expression of C5aR is observed in a number of tumors, including OC, and that C5aR expression strongly and negatively correlates with the levels of CXCL9 and CD8^+^ T cells in cancer patients. Accordingly, cancer patients who display low C5aR expression and high levels of CD8^+^ T cells tend to have a more favorable prognosis. Therefore, our findings highlight the clinical significance of C5aR and its control of CXCL9 in the context of antitumor immunity.

Despite the significant findings presented in this study, several limitations need to be acknowledged. First, the role of macrophage C5aR in tumor control has not been confirmed in the context of macrophage C5aR conditional knockout mice, although the combination of macrophage depletion and C5aR inhibition did not exhibit an additive effect. Second, the mechanisms through which macrophage C5aR contributes to CD8^+^ T cell dysfunction remain unexplored. Additionally, the molecular mechanism through which C5aR deficiency promotes the divergent distribution of Tregs in organs remains unknown. These aspects warrant further investigation in future studies.

## Materials and methods

### Human studies

Human ovarian tissues and sera from patients with epithelial ovarian carcinomas or without ovarian lesions (normal) were acquired from the Affiliated Hospital of Jiangsu University with patient consent. Tumor samples were extracted from patients with primary OC and verified by pathology. Patients with tumor history or other complications were omitted. The experimental design was sanctioned by the Affiliated Hospital of Jiangsu University Ethics Committee (ID: KY2023K0406). The information about the OC samples used is summarized in Table S1.

### Animal studies

For the studies involving animals, appropriate permission was given from the Animal Experimental Ethical Inspection of Chongqing Medical University (ID: IACUC-CQMU-2023-04021). 6- to 8-week-old C57BL/6 (WT) and C57BL/6-background C5aR knockout (C5aR^−/−^) were purchased from Cyagen Biosciences (Shanghai, China) and housed under standard pathogen-free conditions in the animal center of Chongqing Medical University. A 200-μL suspension of 5 × 10^6^ ID8 cells (epithelial OC cell line) were injected intraperitoneally into WT and C5aR^−/−^ female mice on day 0. For studies using αCD4 (clone GK1.5; Bioxcell, West Lebanon, NH, USA), αCD8 (clone 2.43; Bio X Cell, Lebanon, NH, USA), and αCXCL9 (clone MIG-2F5.5; Bio X Cell), antibodies and IgG (clone 2A3; Bio X Cell) were injected intraperitoneally at 200 μg per C5aR^−/−^ mouse on day 30, and every 5 days thereafter until study end. For EdU labeling, 24 h prior to euthanasia, mice were injected intraperitoneally with EdU (K1078, 100 μg/g body weight; APExBIO, Houston, TX, USA). For macrophage depletion studies, C5aR^−/−^ mice were intraperitoneally injected with 200 μL of clodronate liposomes (40335ES05; Yeasen, Shanghai, China) or control liposomes (40338ES05; Yeasen) on day 30 and every 3 days thereafter until completion of the study. PMX-53 is a specific antagonist of C5aR and has been used as a blocker of the C5a-C5aR axis *in vivo* and *in vitro*.^23^ PMX-53 was synthesized by GL Biochem (Shanghai, China) and resuspended in water. For immunotherapy studies, 1 mg/kg PMX-53 or 200 μg of αPD-1 (clone RMP1-14; Bioxcell), alone or combined, was injected into WT mice intraperitoneally on day 30 and every 2 days thereafter for a total of seven injections. For macrophage adoptive transfer studies, 200 μL of suspension of 2 × 10^6^ macrophages extracted from WT or C5aR^−/−^ mice were pretreated for 24 h with TCM containing 100 ng/mL PMX-53 or PBS and then injected intravenously into each mouse on day 30. For T lymphocyte adoptive transfer studies, 200 μL of suspension of 5 × 10^6^ ID8 cells mixed with 1 × 10^6^ T lymphocytes were injected intraperitoneally into WT mice on day 0. The tumor volume was calculated as width^2^ × length/2. On day 60, all mice were sacrificed, and tumor tissues were collected for further analysis.

### IVIS imaging

To monitor tumor progression with the *in vivo* imaging system (IVIS), 5 × 10^6^ luciferase-expressing ID8 (ID8-luc) cells were intraperitoneally injected into WT and C5aR^−/−^ mice. Luciferase signals of bioluminescence were detected with injection of 75 mg/kg D-luciferin potassium (MB1834; Bergolin Biotechnology, Dalian, China) intraperitoneally at days 30, 40, 50, and 60 by the AniView Kirin IVIS Lumina system (Boluteng Biotechnology, Guangzhou, China).

### Cell culture

ID8, a mouse ovarian epithelial cancer cell line widely used to establish a mouse OC model,^55^ was maintained in high-glucose Dulbecco’s modified Eagle’s medium (DMEM, 11995065; Gibco, Carlsbad, CA, USA) supplemented with 10% fetal bovine serum (FBS, 10270-106; Gibco) and 1% penicillin/streptomycin (P/S, 15140122; Gibco) solution. The human OC cell lines SKOV3 and ES2 were maintained in Roswell Park Memorial Institute (RPMI)-1640 medium (22400121; Gibco) supplemented with 10% FBS and 1% P/S. The human myeloid leukemia mononuclear cells (THP1) were maintained in RPMI-1640 supplemented with 10% FBS, 0.05 mM β-mercaptoethanol, and 1% P/S, which can be induced into macrophages by 100 ng/mL phorbol-12-myristate-13-acetate (PMA, 16561-29-8; Sigma, St. Louis, MO, USA). L929 cells are mouse epidermal fibroblasts, which were cultured in DMEM containing 10% FBS and 1% P/S. The supernatant of L929 cells contains a high concentration of M-CSF and can be used to induce differentiation of BMDMs.^56^ All of these cells were purchased from EK Bioscience (Shanghai, China) and cultured at 37°C in a humidified atmosphere containing 5% carbon dioxide. STR profiles match the standards for ID8, SKOV3, ES2, THP1, and L929 cell lines authentication. Mycoplasma testing indicates that the cells used are free of mycoplasma contamination.

### Preparation of BMDMs

Murine macrophages were prepared using BMDMs. Femoral bones were collected from 8- to 10-week-old mice and sterilized in 70% EtOH. The bones were cut open, and the bone marrow was flushed by PBS containing 1% P/S using syringe. Red blood cells were lysed using blood cell lysis buffer (130-094-183; Miltenyi, Cambridge, MA, USA). The cells were then resuspended in PBS and centrifuged over Ficoll-Paque PREMIUM (17544602; Cytiva, Wilmington, DE, USA) to purify the mononuclear cells, subsequently being cultured in DMEM containing 10% FBS, 1% P/S, and 15% L929-conditioned medium for 5 days before being induced in to BMDMs.

### Preparation of PBMC-derived macrophages

PBMCs were isolated from healthy female donors using Histopaque-1077 (10771; Sigma) density gradient centrifugation. Human monocytes were obtained from PBMCs following incubation with CD14 antibody conjugated to magnetic beads (130-050-201; Miltenyi) following the manufacturer’s instructions. The CD14^+^ monocytes were differentiated to macrophages by culturing in 1640 medium containing 10% FBS, 1% P/S, 2 mM L-glutamine, and 100 ng/mL recombinant human M-CSF (SRP3110; Sigma) for 3 days at 37°C in a humidified atmosphere of 5% CO_2_.

### Macrophage polarization and stimulation

BMDMs, PBMC-derived macrophages, and THP1-derived human macrophages were polarized toward M1 phenotype by either 100 ng/mL IFN-γ (AF-300-02; Peprotech, Rocky Hill, NJ, USA) plus 50 ng/mL LPS (L2880; Sigma) or LPS alone for 24 h, or M2 phenotype by a combination of 40 ng/mL IL-4 (AF-200-04; Peprotech) and 40 ng/mL IL-10 (210-10; Peprotech) treatment for 24–48 h. To mimic TAMs *in vitro*, naive macrophages were stimulated with TCM, and macrophages were cultured with complete medium containing 50% TCM (volume) for 24 h. For C5aR antagonist studies, PMX-53 (100 ng/mL) or PBS was incubated with TAMs for 2 h. For inhibitor studies, 10 μM Erk inhibitor FR180204 (S7524; Selleck, Houston, TX, USA), 10 μM Akt inhibitor GSK2141795 (S7492; Selleck), or 10 μM NF-κB/p65 inhibitor pyrrolidinedithiocarbamate ammonium (S3633; Selleck) were incubated with TAMs for 24 h after the addition of 100 ng/mL PMX-53 stimulation.

### Isolation and culture of splenic T lymphocytes

Spleens were collected from 8- to 12-week-old WT mice under aseptic conditions, followed by mechanistic disruption through a 70-μm cell strainer (130-098-462; Miltenyi) into a single-cell suspension. After lysing red blood cells, splenocytes were inoculated on the plate coated with 0.5 μg/mL anti-CD3 (clone 145-2C11; BioLegend, San Diego, CA, USA) and 5 μg/mL anti-CD28 (clone 37.51; BioLegend) antibodies in RPMI-1640 medium supplemented with 50 mM β-mercaptoethanol (60-24-2; Millipore, Boston, MA, USA) and 10 mM HEPES (7365-45-9; Millipore) and activated for 72 h.

### *In vitro* co-culture assay

To analyze the effect of C5aR-deficient macrophages on ID8 cells, WT BMDMs or C5aR^−/−^ BMDMs and the ID8 cell line were co-cultured using a non-contact co-culture transwell system (Corning, Corning, NY, USA). BMDMs pretreated with TCM were seeded in 0.4-μm pores (1 × 10^5^ cells per pore), and 24-well plates were seeded with ID8 cells (1 × 10^5^ cells per well). We added 100 ng/mL PMX-53 or PBS into WT or C5aR^−/−^ BMDMs. After 48 h of co-culture, the ID8 cells were harvested for the detection of apoptosis, including Annexin V/PI by flow cytometry and TUNEL stain. To analyze the effect of C5aR-deficient macrophages on the migration of T lymphocytes, T lymphocytes labeled with 10 μM CM-DiI (40718ES50; Yeasen) were seeded in 5-μm pores (1 × 10^5^ cells per pore), and 24-well plates were seeded with TCM-pretreated WT or C5aR^−/−^ BMDM (1 × 10^5^ cells per well) or containing basal medium or TCM without BMDM seeding. Respectively, 100 ng/mL αCXCL9 or IgG was incubated with C5aR^−/−^ BMDMs or added into TCM for 3 h, then T lymphocytes migrating to the lower layers were photographed using fluorescent microscopy. To analyze the effect of C5aR-deficient macrophages on the cytotoxicity of CD8^+^ T cells, T lymphocytes were mixed 1:1 with WT BMDMs or C5aR^−/−^ BMDMs. PMX-53 (100 ng/mL) or PBS was added to the medium and, after incubation for 48 h, cells were analyzed for the ratio of CD8^+^IFN-γ^+^ and CD8^+^perforin^+^ by FCM.

### TCGA data mining

The TIMER2.0 website (http://timer.cistrome.org) was used to analyze the relevance of *C5aR* and *CXCL9* mRNA level with the expression of immune-related genes or immune cell infiltration in human OC samples based on TCGA database of RNA-seq data. The GEPIA website (http://gepia.cancer-pku.cn) is an interactive website based on data from TCGA and GTEx projects, which was accessed to analyze the expression of *C5aR* and *CXCL9* in various kinds of cancer tissues and normal tissues. Log_2_ (TPM +1) transformed expression data were chosen for plotting. For overall survival analysis based on *C5aR* and *CXCL9* gene expression, patients were divided into either a high-expression group or a low-expression group using k-means 2 separation of gene expression values (consensus from 20 iterations). The log-rank test was used for the analysis of overall survival.

### RNA sequencing

ID8 cells were intraperitoneally injected into WT or C5aR^−/−^ mice. Two months later, tumors were harvested and collected and single-cell suspension was generated following the methods described below in “flow-cytometry analysis.” Live CD45^+^CD11b^+^Ly6G^−^F4/80^+^ TAM (10^6^) were sorted by flow cytometry and submitted to Shanghai Majorbio Bio-pharm Technology for sequencing. Total RNA from WT or C5aR^−/−^ TAM was extracted and purified using TRIzol reagent (Invitrogen, Carlsbad, CA, USA) following the manufacturer’s procedure. Poly(A) RNA was purified from 1 μg of total RNA using Dynabeads Oligo(dT) 25-61005 (Thermo Fisher, Waltham, MA, USA) using two rounds of purification. The poly(A) RNA was then fragmented into small pieces using Magnesium RNA Fragmentation Module (NEB, Ipswich, MA, USA). The cleaved RNA fragments were reverse-transcribed to create the cDNA by SuperScript II Reverse Transcriptase (Invitrogen), which were next used to synthesize U-labeled second-stranded DNAs with *E. coli* DNA polymerase I (NEB), RNase H (NEB), and dUTP Solution (Thermo Fisher). After the heat-labile UDG enzyme (NEB) treatment of the U-labeled second-stranded DNAs, the ligated products were amplified with PCR. The average insert size for the final cDNA library was 300 ± 50 bp. Lastly, we performed 2 × 150 bp paired-end sequencing (PE150) on an Illumina Novaseq 6000 (LC-Bio Technologies, Hangzhou, China) following the vendor’s recommended protocol.

### Bioinformatics analysis of RNA-seq

Fastp software was used to remove the reads that contained adaptor contamination, low-quality bases, and undetermined bases with default parameters. We used HISAT2 to map reads to the reference genome. The mapped reads of each sample were assembled using StringTie with default parameters. All transcriptomes from all samples were then merged to reconstruct a comprehensive transcriptome using gffcompare. After the final transcriptome was generated, StringTie and was used to estimate the expression levels of all transcripts. StringTie was used to perform expression level for mRNAs by calculating FPKM (fragments per kilobase per million mapped reads). The differentially expressed mRNAs were selected with fold change >2 or fold change <0.5 and with parametric F test comparing nested linear models (p value <0.05) by R package edgeR. Gene differential expression analysis was performed by DESeq2 software between two different groups (and by edgeR between two samples). The genes with the parameter of false discovery rate <0.05 and absolute fold change ≥2 were considered differentially expressed genes. Differentially expressed genes were then subjected to enrichment analysis of gene ontology functions and Kyoto Encyclopedia of Genes and Genomes (KEGG) pathways using the free online platform of Majorbio Cloud Platform (www.majorbio.com).

### Flow-cytometry analysis

ID8 tumors were digested using the Tumor Dissociation Kit (130-095-929; Miltenyi) and processed to generate single-cell suspensions by dissociator (130-093-235; Miltenyi), as described in the instructions. The cell suspensions were filtering through a 70-μm cell strainer and centrifuged at 500 × *g* for 5 min. Cell pellets were pretreated with blood cell lysis buffer and incubated with TruStain FcXTM (1:100) (422301; BioLegend) for 20 min on ice. Dead cells were excluded using the Fixable Viability Kit (564996; BD Pharmingen, San Jose, CA, USA). All subsequent antibodies were stained for 20 min on ice, and were purchased from BioLegend. For myeloid cell analysis, APC/Cy7 anti-CD45 (clone 30-F11), BV510 anti-CD11b (clone M1/70), PerCP/Cy5.5 anti-Ly6G (clone 1A8), PE anti- Ly-6G/Ly-6C (Gr-1) (clone RB6-8C5), BV421 anti-CD11c (clone 418), APC anti-F4/80 (clone BM8), FITC anti-CD86 (clone PO3), PE anti-CD206 (clone C068C2), PE anti-CXCL9 (clone MIG-2F5.5), PE/Cy7 anti-C5aR (clone 20/70), BV421 anti-MHCII (clone M5/114.15.2), PerCP/Cy5.5 anti-IL-10 (clone JES5-16E3), PE anti-TNF-α (clone TN3-19.12), PE/Cy7 anti-CD163 (clone S15049F), PE anti-iNOS (clone W16030C), PE anti-TGF-β1 (clone TW7-20B9), and PerCP/Cy5.5 anti-IL-4 (clone 11B11) were used for different panels. For T lymphocyte analysis, APC/Cy7 anti-CD45, PE/Cy7 anti-CD3 (clone 17A2), APC anti-NK1.1 (clone S17016D), PerCP/Cy5.5 anti-CD4 (clone RM4-4), FITC anti-CD8 (clone 53-6.7), APC anti-CD25 (clone QA19A49), PE anti-FoxP3 (clone MF-14), Pacific Blue anti-granzyme B (clone GB11), PE anti-IFN-γ (clone XMG1.2), APC anti-perforin (clone S16009A), PerCP/Cyanine5.5 anti-LAG-3 (clone C9B7W), APC anti-PD1 (clone RMP1-30), PE anti-CLTA-4 (clone UC10-4F10-11), APC anti-TOX (568356; BD Biosciences), PerCP/Cyanine5.5 anti-Tim-3 (clone RMT3-23), and BV421 anti-SLAMF6 (566692; BD Biosciences) were used. Lymphocytes (CD45^+^), neutrophils (CD45^+^, CD11b^+^, F4/80^−^, Ly6G^+^), DCs (CD45^+^, CD11c^+^), MDSCs (CD45^+^, CD11b^+^, F4/80^+^, Gr1^+^), macrophages (CD45^+^, CD11b^+^, Ly6G^−^, F4/80^+^), M1-like cells (CD45^+^, CD11b^+^, Ly6G^−^, F4/80^+^, CD86^+^), M2-like cells (CD45^+^, CD11b^+^, Ly6G^−^, F4/80^+^, CD206^+^), CD4^+^ T cells (CD45^+^, CD3^+^, CD4^+^), CD8^+^ cells (CD45^+^, CD3^+^, CD8^+^), Tregs (CD45^+^, CD3^+^, CD4^+^, CD25^+^, FoxP3^+^), and NK cells (CD45^+^, CD3^−^, NK1.1^+^) were gated according to the corresponding markers (Figure S10). The Transcription Factor Staining Buffer Set (88-8824-00; eBioscience, San Diego, CA, USA) was used for FoxP3 staining. For intracellular cytokine staining, the samples were preincubated with GolgiStop (1:1,000) (554715; BD Biosciences) and permeabilized by the intracellular permeabilization wash buffer (BioLegend) following the manufacturer’s instructions. To identify apoptotic and necrotic tumor cells co-cultured with macrophages, an FITC Annexin V apoptosis detection kit (40302ES50; Yeasen) with propidium iodide was used. For the EdU assay, EdU incorporation was detected by the CY5 EdU Staining Kit for Flow Cytometry (K1078; APExBIO). To confirm the reach of adoptive transferred macrophages into the *in situ* tumors of host mice, we stained cells with PKH67 (D0031; Solarbio, Beijing, China) according to the instructions and examined the proportion of PKH67^+^ macrophages in the tumors of mice after receiving PKH67-labeled macrophages for 10 days. Data acquisition was performed on an FACS AriaIII Flowmeter (BD Biosciences), and FlowJo software (version V10; Becton Dickinson, Ashland, OR, USA) was used for analysis.

### ELISA assays

Tumors from WT and C5aR^−/−^ mice were homogenized in iced PBS containing complete EDTA-free protease inhibitors (P1049; Beyotime, Shanghai, China) and 1.0 mM phenylmethylsulfonyl fluoride. Homogenates, supernatants, and sera were centrifuged at 12,000 rpm and 4°C and stored at −20°C for analysis. Serum of patients and mice, supernatants of macrophage and OC cell lines, and protein lysate from ID8 tumors were used in ELISA to detect C3a (MB-3138B; Meibiao Biology, Yancheng, China), C5a (MB-3137A/ED-19918; Meibiao Biology), TNF-α (KE10002/KE00154; Proteintech, Wuhan, China), IFN-γ (KE10001/KE00146; Proteintech), and CXCL9 (KE00165/KE10067; Proteintech) according to the manufacturer’s instructions.

### RNA extraction and RT-qPCR analysis

The RT-qPCR assay of human OC and normal ovarian tissues, ID8 tumors, and cells was conducted as previously described.^57^ Total RNA was extracted by RNAiso Plus (9108; Takara, Kyoto, Japan), and single-stranded cDNA was synthesized with the PrimeScript RT Master Mix kit (RR014; Takara). Relative mRNA levels of antioxidant genes were determined by the Bio-Rad Real-Time PCR System with TB Green Premix Ex Taq (RR420; Takara). The expression values were normalized to the average CT value of GAPDH. All primers used for RT-qPCR are listed in Table S2.

### Protein extraction and western blotting

Proteins from human tissues and different groups of macrophages or tumor cells were isolated by homogenizing in a RIPA buffer (P0013B; Beyotime) with protease inhibitors. After measuring the total protein concentration using a BCA protein kit (P0009; Beyotime), the proteins were separated by electrophoresis and transferred to polyvinylidene fluoride membranes. After blocking with 5% skimmed milk at room temperature for 2 h, the membrane was incubated with 1:1,000 dilution of anti-C5aR (21316-1-AP; Proteintech), anti-CXCL9 (22355-1-AP; Proteintech), anti-p-Erk (28733-1-AP; Proteintech), anti-Erk (11257-1-AP; Proteintech), anti-p-Akt (66444-1-Ig; Proteintech), anti-Akt (60203-2-Ig; Proteintech), anti-p-NF-κB/p65 (3033T; Cell Signaling Technology, Danvers, MA, USA), anti-NF-κB/p65 (80979-1-RR; Proteintech), anti-p-C/EBPβ (AP1055; ABclonal, Wuhan, China), anti-C/EBPβ (A19538; ABclonal), anti-p-STAT1 (AF330; ABclonal), anti-STAT1 (10144-2-AP; Proteintech), anti-p-STAT6 (AF3301; ABclonal), anti-STAT6 (51073-1-AP; Proteintech), or anti-β-actin (81115-1-RR; Proteintech) overnight. Thereafter it was washed and incubated with the corresponding secondary antibody (Proteintech) for 2 h. A chemiluminescent kit was used to detect the antibody-bound protein. Blots were detected by a chemiluminescent kit, and quantitative analysis was performed by ImageJ software. The protein expression level was normalized to β-actin.

### Immunohistochemical staining

Immunohistochemical staining was performed according to standard procedures. Sections were incubated with C5aR or CD8 primary antibody at a 1:500 dilution overnight at 4°C. After washing with PBS, 100 μL of secondary antibody was added to each slice before incubation at 4°C again for 50 min. The negative control used PBS instead of primary antibody. All slices were then counterstained with hematoxylin, dehydrated through graded alcohol, and cemented with neutral gum. False-positive immunohistochemical staining was excluded, and each slice that was non-overlapping was randomly selected. ImageJ software was used to measure the average optical density (AOD) of positive signal per field.

### Immunofluorescence staining and TUNEL assay

After deparaffinizing and rehydrating, antigen retrieval was carried out as previously described using proteinase K for 30 min. After antigen retrieval, tissue or cell sections were incubated with 5% bovine serum albumin (BSA, ST023; Beyotime) for 30 min at room temperature. The tissues were then incubated with primary antibodies in 5% BSA/PBS overnight at 4°C. The following primary antibodies were used at a dilution of 1:500: C5aR, CXCL9, pan-keratin (CK, 26411-1-AP; Proteintech), CD11C (17342-1-AP; Proteintech), CD66b (A8113; ABclonal), CD56 (14255-1-AP; Proteintech), CD3 (17617-1-AP; Proteintech), CD68 (28058-1-AP; Proteintech), CD80 (66406-1-Ig; Proteintech), CD206 (18704-1-AP; Proteintech), F4/80 (29414-1-AP; Proteintech), CD11b (66519-1-Ig; Proteintech), CD4 (67786-1-Ig; Proteintech), CD8 (66868-1-Ig; Proteintech), IFN-γ (15365-1-AP; Proteintech), and granzyme B (252579; ZenBio, Durham, NC, USA). Secondary Alexa Fluor 488 and 594 antibodies were purchased from Proteintech and used at 1:1,000 dilution factor. Co-staining was performed using a multiple immunofluorescence kit (AFIHC026; Hunan Aifang Biological Technology, China) based on the tyramide signal amplification technology according to the manufacturer’s instructions. For TUNEL staining, rather than primary and secondary antibody incubation on each slide, equilibration buffer was incubated for 30 min followed by fluorescein-labeled solution with TdT enzyme (A111-01; Vazyme, Nanjing, China) and incubated for 60 min at 37°C in a moist, dark chamber. All of the slides were washed and incubated for 10 min with Hoechst 33342 (1:1,000) (CA1120; Solarbio, Beijing, China). Slides of tissue and cell samples were photographed by an Olympus fluorescence microscope, and the ratio of positive cells was calculated by ImageJ software.

### Statistical analysis

Prism 9.0 software (GraphPad, La Jolla, CA, USA) was used for statistical analysis and making graphs. Statistical differences between the two groups were determined using parametric or non-parametric Student’s t test, and one-way or two-way ANOVA was performed for multiple comparisons. A p value of <0.05 was considered statistically significant (∗p < 0.05, ∗∗p < 0.01, ∗∗∗p < 0.001, ∗∗∗∗p < 0.0001 in figures). Survival was evaluated using the Kaplan-Meier method and analyzed by the Mantel-Cox log-rank test. All experiments were performed at least three times independently.

## Data and code availability

RNA-seq datasets have been deposited in the Gene Expression Omnibus under accession code GEO: GSE243210. All raw data supporting the findings are available from the corresponding authors upon reasonable request.

## Supplemental information

Document S1. Figures S1–S10 and Tables S1 and S2

Document S2. Article plus supplemental information
