# Supplementary material for: Blockade of C5a receptor unleashes tumor-associated macrophage antitumor response and enhances CXCL9-dependent CD8+ T cell activity
Source: Mol Ther. 2023 Dec 14;32(2):469–89. doi: 10.1016/j.ymthe.2023.12.010 (PMC10861991; doi:10.1016/j.ymthe.2023.12.010)
Supplement: Document S1. Figures S1–S10 and Tables S1 and S2 [file mmc1.pdf]

YMTHE, Volume *32*

Supplemental Information

Blockade of C5a receptor unleashes tumor-associated macrophage antitumor response and enhances CXCL9-dependent CD8$^+$ T cell activity

Xiaojin Luan, Ting Lei, Jie Fang, Xue Liu, Huijia Fu, Yiran Li, Wei Chu, Peng Jiang, Chao Tong, Hongbo Qi, and Yong Fu

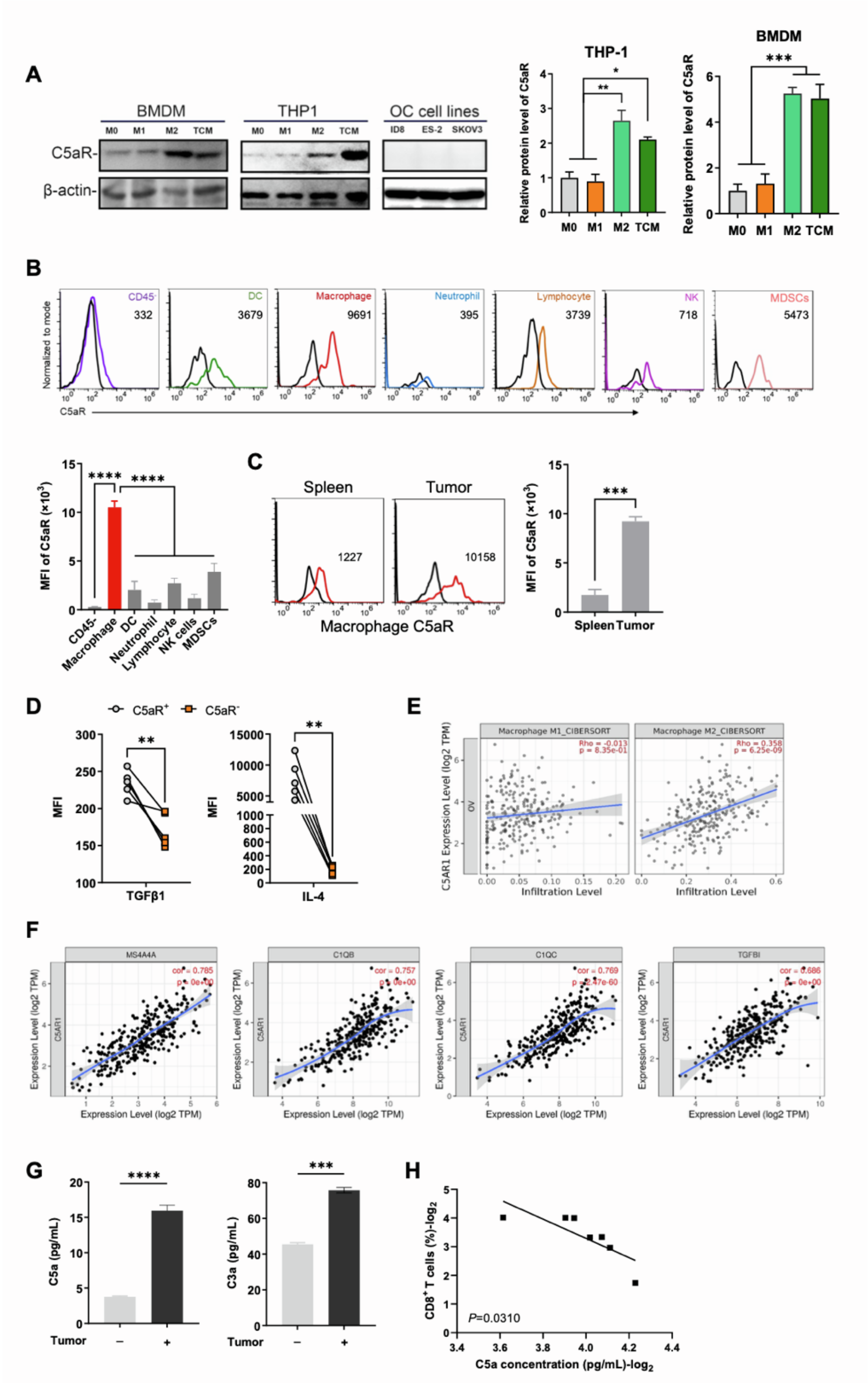

Fig.S1 C5aR is highly expressed on tumor-associated macrophages (related to Fig.1)

A Representative western blot analysis of C5aR expression in human and mouse OC cell lines and different groups of THP1-derived macrophage and BMDM. β-actin was used as a loading control (n=3 independent replicates; one-way ANOVA). **B** Flow cytometry analysis of C5aR expression on $CD45^-$ cells, DC, macrophages, neutrophil, lymphocyte, NK and MDSCs in ID8 tumors from WT mice (n = 3 mice; one-way ANOVA; representative of three independent experiments). **C** Flow cytometry analysis of *C5aR* expression on spleen- or tumor-infiltrating macrophages (n = 3 mice; two-tailed paired *t* test; representative of three independent experiments). **D** Flow cytometry analysis of TGFβ1 and IL-4 expression on $C5aR^+$ or $C5aR^-$ macrophages in ID8 tumors (n = 4 mice; two-tailed paired *t* test). **E** Analysis of correlations between *C5aR* expression and M1- or M2-macrophages based on the RNA-seq results from TCGA database of ovarian cancers using TIMER. The corrected partial Spearman's correlation coefficient and statistical *P* value were presented. **F** Analysis of correlations between *C5aR* expression and M2-like macrophages related markers (*MS4A4A*, *C1QB*, *C1QC* and *TGFB1*) based on the RNA-seq results from TCGA database of ovarian cancers using TIMER. The corrected partial Spearman's correlation coefficient and statistical *P* value were presented. **G** Serums of ID8 tumor-free and tumor-bearing mice were harvested for quantifying C3a and C5a production by ELISA assay (n = 3 mice; two-tailed unpaired *t* test). **H** Pearson correlation between C5a concentration and tumor-infiltrating $CD8^+$ T cells measured by flow cytometry expression (n=7 mice). The statistical *P* value were presented. Data are represented as the mean ± SEM. *$p < 0.05$, **$p < 0.01$, ***$p < 0.001$. ****$p < 0.0001$, ns represents no significance.

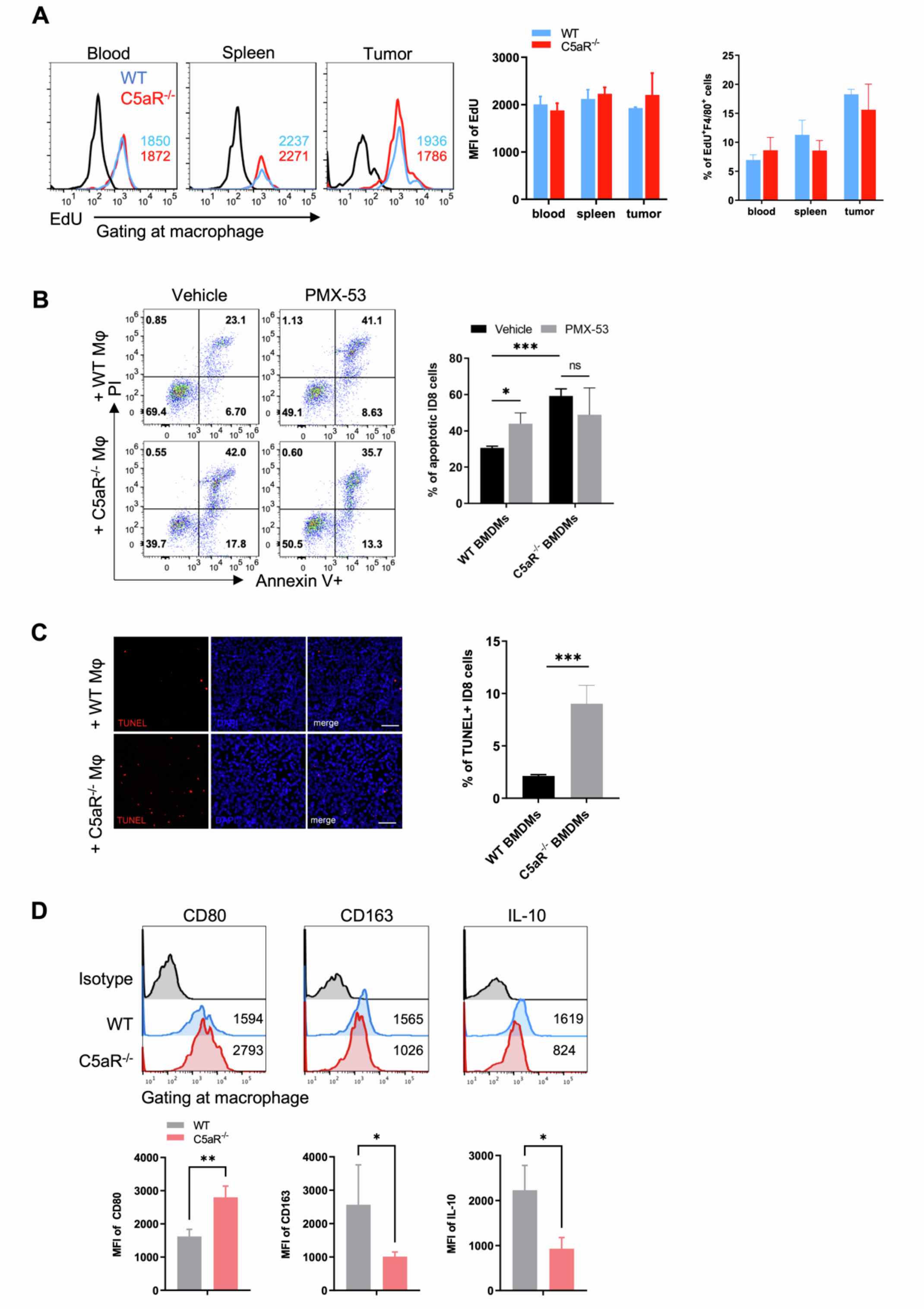

Fig.S2 C5aR deficiency enhances the antitumor capacity of TAM (related to Fig.2)
A Flow cytometry analysis of EdU expression on spleen- or tumor-infiltrating macrophages (n = 3 mice; two-way ANOVA). **B** Flow cytometry analysis of annexin V/PI staining and quantification of ID8 cells cocultured with WT BMDMs or C5aR$^{-/-}$ BMDMs in TCM with or without PMX-53 (n=3 independent replicates; two-way ANOVA). **C** Representative TUNEL-stained ID8 cells cocultured with WT BMDMs or C5aR$^{-/-}$ BMDMs. The proportion of TUNEL-positive cells was qualified from five random fields, representing the apoptotic index. (n=3 independent replicates; two-tailed unpaired *t* test). Scale bars, 100 μm. **D** Flow cytometry analysis of CD80, CD163 and IL-10 expression on tumor-infiltrating macrophages from WT and C5aR$^{-/-}$ tumor bearing mice (n = 3 mice; two-tailed unpaired *t* test). Results are representative of three independent experiments. Data are represented as the mean ± SEM. $^*p < 0.05$, $^{**}p < 0.01$, $^{***}p < 0.001$. $^{****}p < 0.0001$, ns represents no significance.

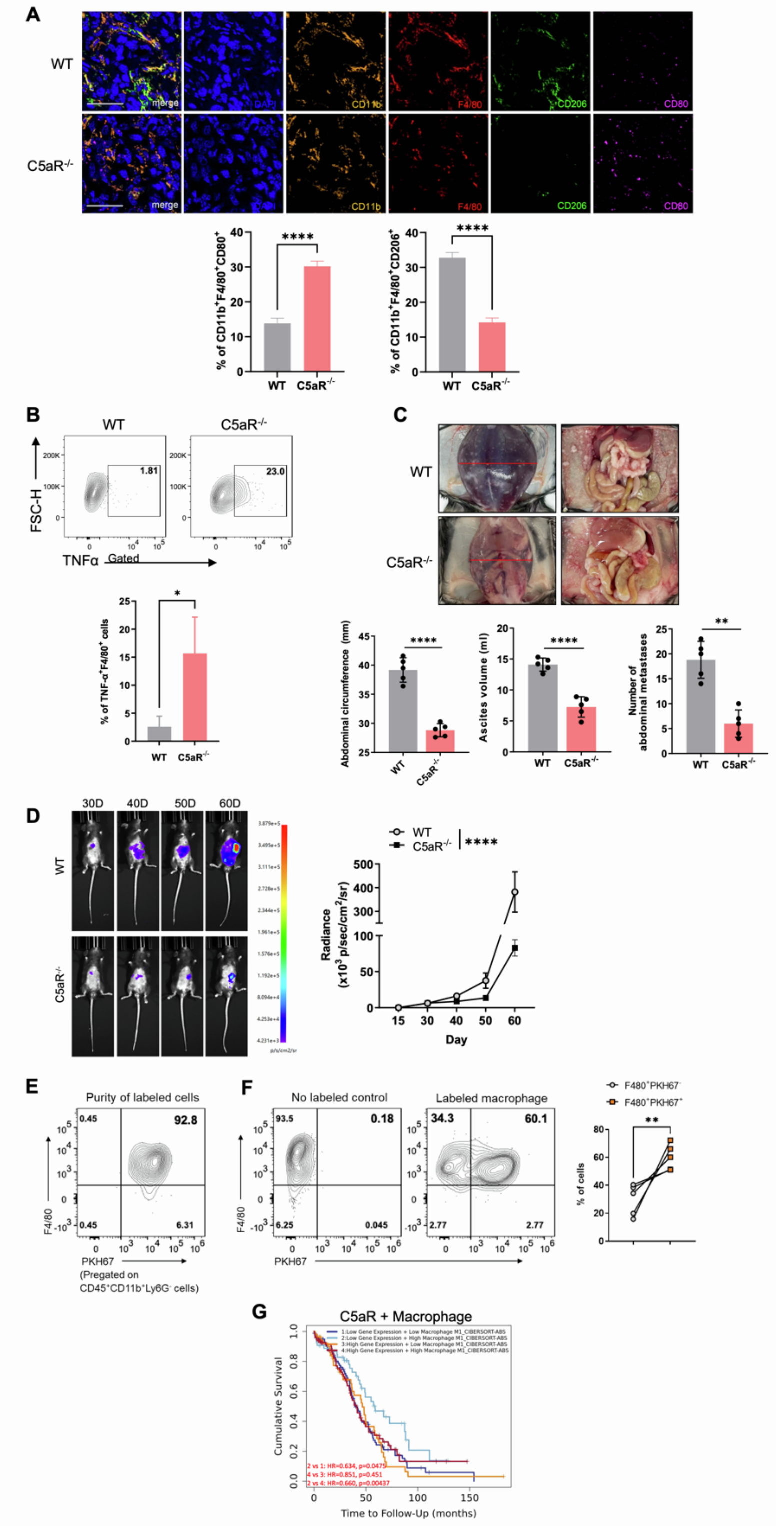

Fig.S3 C5aR deficiency promotes M1-like TAM polarization and limits tumor growth (related to Fig.2)

A Representative photomicrographs and quantitative analysis of proportion of $CD11b^+F4/80^+CD80^+$ and $CD11b^+F4/80^+CD206^+$ cells in ID8 tumors from WT and $C5aR^{-/-}$ mice (n = 3 mice; two-tailed unpaired *t* test). Scale bars, 50 μm. **B** Proportions of $TNF\alpha^+$ cells among $F4/80^+$ TAM from WT and $C5aR^{-/-}$ mice were measured using flow cytometry (n = 3 mice; two-tailed unpaired *t* test). **C** Abdominal circumference, ascites volume and the number of abdominal metastases were measured on day 60 post ID8 tumor into WT and $C5aR^{-/-}$ mice (n = 3 mice; two-tailed unpaired *t* test). **D** Representative live animal images and quantification of ID8-luc models at day 30, 40, 50, 60 in WT and $C5aR^{-/-}$ mice (n = 3 mice at each time point; two-tailed unpaired *t* test). **E** Proportions of $PKH67^+$ BMDM were measured using flow cytometry. **F** Proportions of $PKH67^+$ TAM of tumor-bearing WT and $C5aR^{-/-}$ mice receiving PKH67-labeled BMDM. **G** Kaplan–Meier analysis of the relationship between the *C5aR* mRNA expression level in synergy with the degree of macrophage tumor infiltration and prognosis of ovarian cancer patients from the TCGA dataset. *p* value showed the statistical difference by log-rank test. Results are representative of three independent experiments. Data are represented as the mean ± SEM. *$p < 0.05$, **$p < 0.01$, ***$p < 0.001$. ****$p < 0.0001$, ns represents no significance.

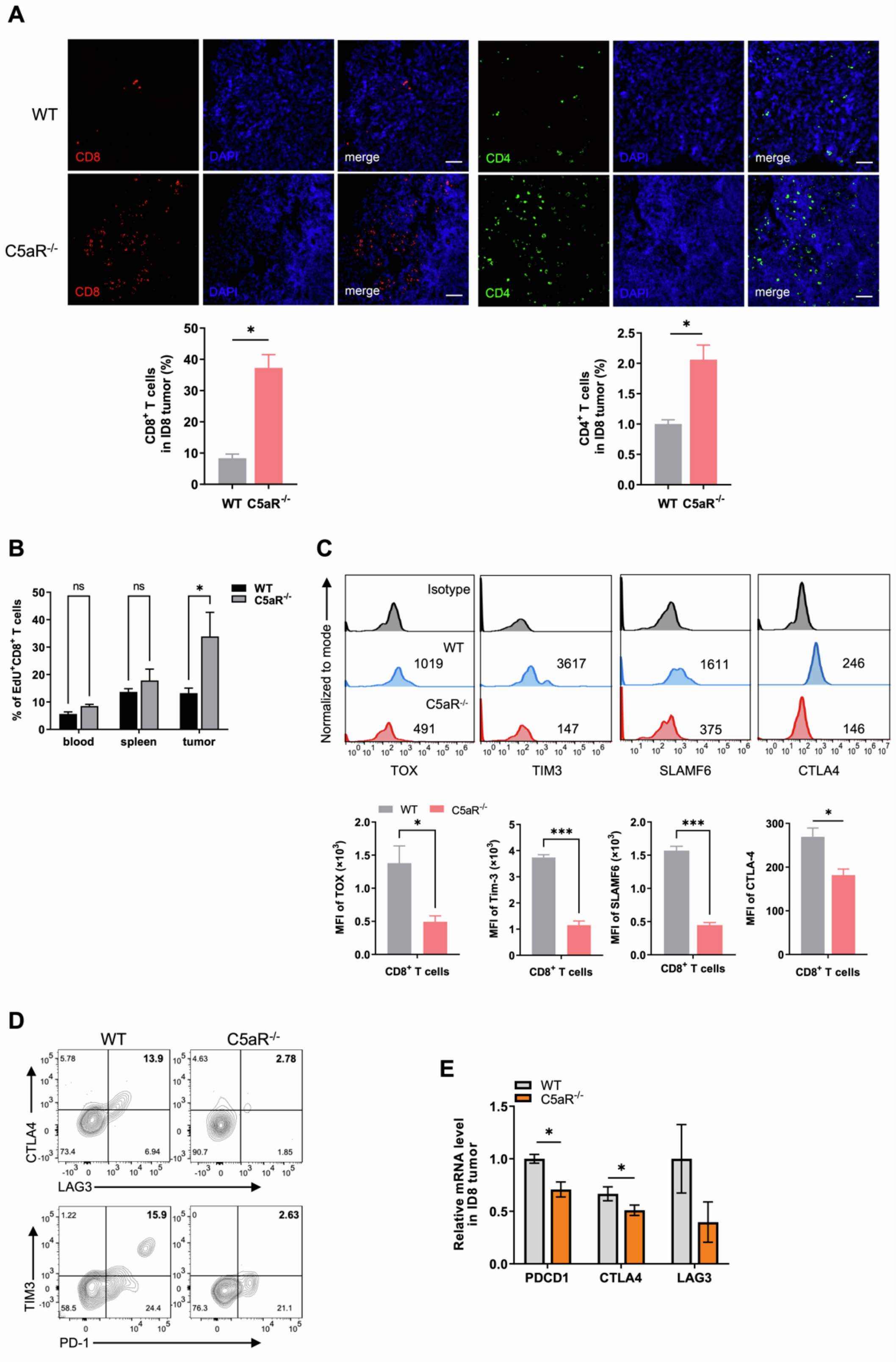

Fig.S4 C5aR deficiency decreases exhausted CD8^{+} T cell in TME (related to Fig.3)

A Representative photomicrographs and quantitative analysis of ID8 tumors immunofluorescent staining of CD8 (red), CD4 (green), and DAPI (blue) from WT and C5aR$^{-/-}$ mice (n = 3 mice; two-tailed unpaired *t* test). Scale bars, 50 μm. **B** Proportion of peripheral blood, spleen and tumor-infiltrating EdU^{+}CD8^{+} T cells measured by flow cytometry (n = 3 mice; two-way ANOVA). **C** Flow cytometry analysis of expression of TOX, Tim-3, SLAMF6, CTLA-4 on CD8^{+} T cells from WT and C5aR$^{-/-}$ mice (n = 3 mice; two-tailed unpaired *t* test). **D** Proportions of PD-1^{+}TIM3^{+} and LAG-3^{+}CTLA4^{+} cells of tumor-infiltrating CD8^{+} T cells from WT and C5aR$^{-/-}$ mice were measured using flow cytometry (n = 3 mice). **E** Relative mRNA of exhausted T cell related genes (*PDCD1*, *CTLA4*, and *LAG3*) expression level in ID8 tumors from WT and C5aR$^{-/-}$ mice. (n = 3 mice; two-way ANOVA). Results are representative of three independent experiments. Data are represented as the mean ± SEM. *$p < 0.05$, **$p < 0.01$, ***$p < 0.001$. ****$p < 0.0001$, ns represents no significance.

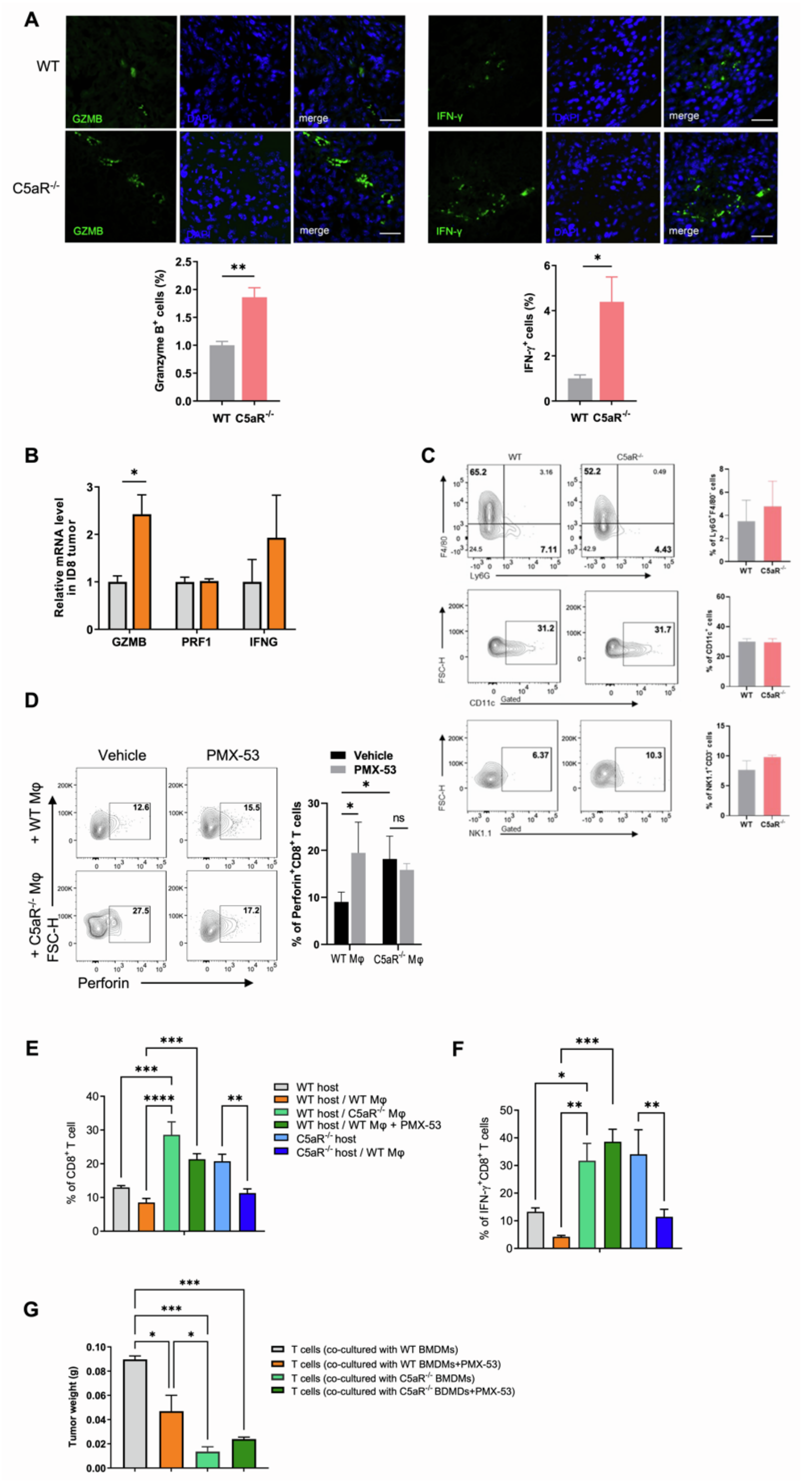

Fig.S5 C5aR deficiency enhances cytotoxicity of CD8$^+$ T cells (related to Fig.3)

A Representative photomicrographs and quantitative analysis of ID8 tumors immunofluorescent staining of GranzymeB (GZMB; green), IFN-γ (green) and DAPI (blue) from WT and C5aR$^{-/-}$ mice (n = 3 mice; two-tailed unpaired *t* test). Scale bars, 50 μm. **B** Relative mRNA of cytotoxic T cell related genes (*GZMB*, *PRF1*, and *IFNG*) expression level in ID8 tumors from WT and C5aR$^{-/-}$ mice. (n = 3 mice; two-way ANOVA). **C** Proportion of neutrophil (Ly6G$^+$F4/80$^-$), DC (CD11c$^+$) and NK (NK1.1$^+$CD3$^-$) cells measured by flow cytometry on day 60 after ID8 tumor inoculation in WT and C5aR$^{-/-}$ mice (n = 3 mice; two-tailed unpaired *t* test). **D** Proportion of Perforin$^+$CD8$^+$ T cells of murine splenic lymphocytes cultured mixed with WT BMDMs or C5aR$^{-/-}$ BMDMs (1:1 ratio) in TCM with or without PMX-53 was measured by flow cytometry (n=3 independent replicates; two-way ANOVA). **E-F** Proportion of CD8$^+$ T (**E**) and IFN-γ$^+$CD8$^+$ T cells (**F**) in TME of tumor-bearing WT and C5aR$^{-/-}$ mice receiving BMDM (2×10^6 Mφ; i.v.) from WT or C5aR$^{-/-}$ mice with or without PMX-53 (100 ng/mL) treatment was measured by flow cytometry (n = 3 mice; one-way ANOVA). **G** Weight of tumors on day 60 from WT mice seeded with a mixture of T cells and ID8 cells (1:5 ratio) in suspension (n = 3 mice; one-way ANOVA). T cells were pre-cocultured with WT or C5aR$^{-/-}$ macrophages with or without PMX-53 (100 ng/mL). Results are representative of three independent experiments. Data are represented as the mean ± SEM. *$p < 0.05$, **$p < 0.01$, ***$p < 0.001$. ****$p < 0.0001$, ns represents no significance.

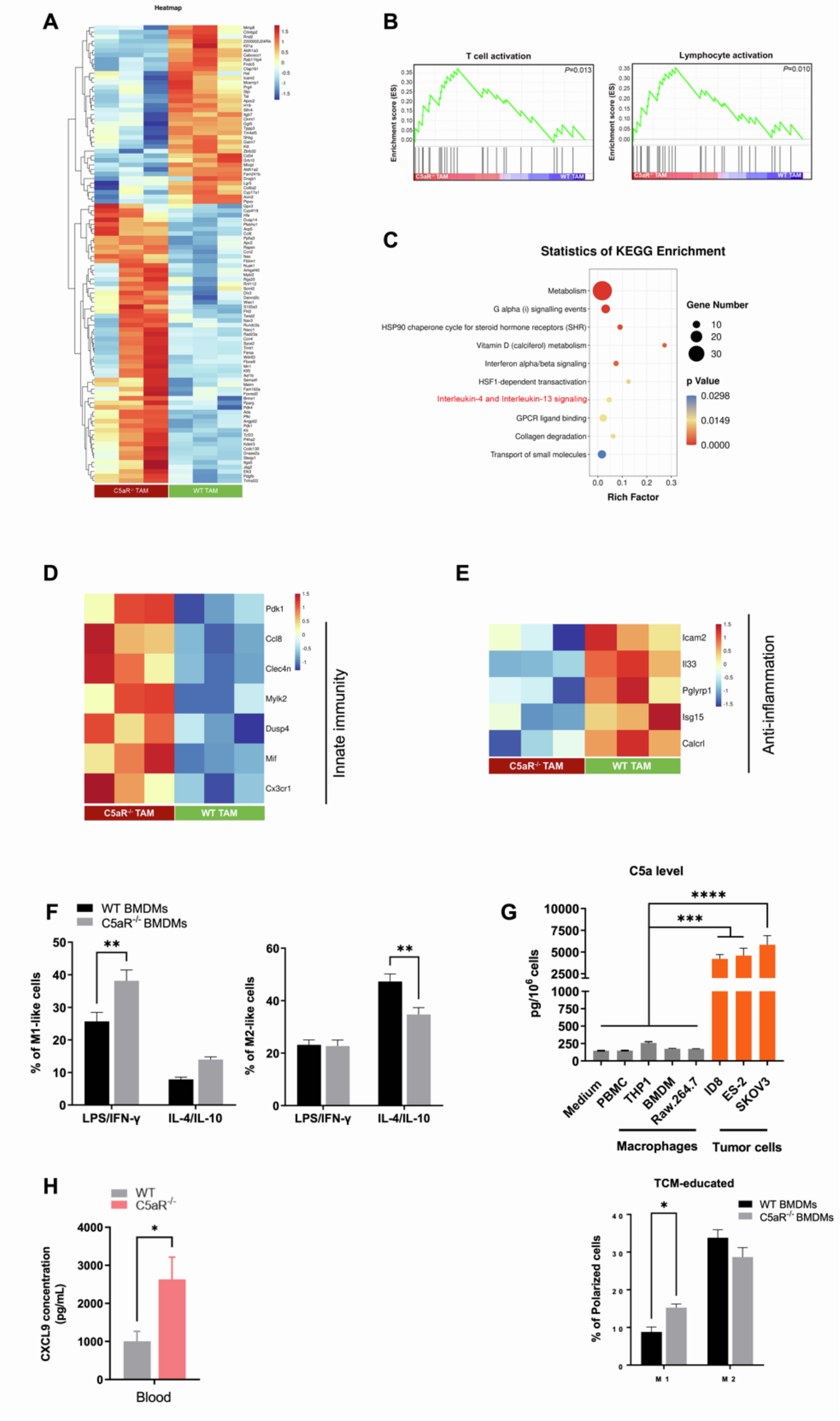

Fig.S6 C5aR deletion alters the transcriptional landscape of macrophages (related to Fig.4)

A Heatmap of RNA-seq data showing the up- and downregulation genes between WT or C5aR$^{-/-}$ TAM (n=4 mice per group). **B** Gene Set Enrichment Analysis (GSEA) analysis of differential genes between WT or C5aR$^{-/-}$ TAM. Statistical *P* value were presented. **C** KEGG enrichment analysis of down-regulated differentially expressed genes between WT or C5aR$^{-/-}$ TAM. **D-E** Heatmap depicting relative expression of innate immunity (**D**) or anti-inflammation (**E**) related genes in differentially expressed genes between WT or C5aR$^{-/-}$ TAM. **F** Proportions of M1-like or M2-like cells among WT or C5aR$^{-/-}$ BMDM stimulated by IFNγ (100 ng/mL) plus LPS (50 ng/mL) or IL-4 (40 ng/mL) plus IL-10 (40 ng/mL) were measured using flow cytometry (n=3 independent replicates; one-way ANOVA). **G** C5a level in basal medium and medium of macrophages or OC cell lines was detected by ELISA (above; n=3 independent replicates; two-way ANOVA). Proportions of M1-like or M2-like cells among WT or C5aR$^{-/-}$ BMDM stimulated by TCM were measured using flow cytometry (below; n=3 independent replicates; two-way ANOVA). **H** Serums of ID8 tumor-bearing WT or C5aR$^{-/-}$ mice were harvested for quantifying CXCL9 production by ELISA assay (n = 5 mice; two-tailed unpaired *t* test). Data are represented as the mean ± SEM. $^{*}p < 0.05$, $^{**}p < 0.01$, $^{***}p < 0.001$. $^{****}p < 0.0001$, ns represents no significance.

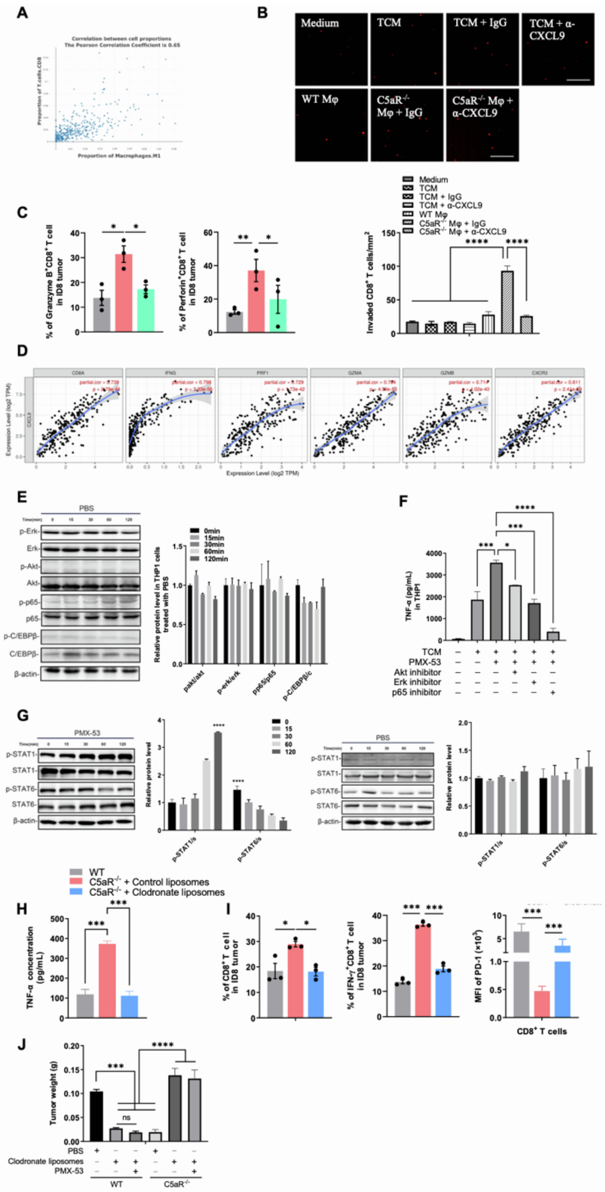

Fig.S7 Loss of C5aR promotes infiltration of CD8$^+$ T cells mediated by TAM-secreted CXCL9 (related to Fig.5)

A Association analysis between proportion of tumor-infiltrating CD8$^+$T cells and M1 macrophages based on the RNA-seq results from TCGA database. The Pearson correlation coefficient was presented. **B** Representative photomicrographs (above) and quantitative analysis (below) of migrated CD8$^+$T cells (n=3 independent replicates; one-way ANOVA). Scale bars, 100 µm. **C** Proportion of tumor-infiltrating GranzymeB$^+$CD8$^+$ T and Perforin$^+$CD8$^+$ T cells from WT and C5aR$^{-/-}$ mice receiving αCXCL9 or IgG treatment was measured by flow cytometry (n = 3 mice; one-way ANOVA). **D** Analysis of correlations between CXCL9 expression and cytotoxic CD8$^+$ T lymphocytes related markers (*CD8A*, *IFNG*, *PRF1* and *GZMA*) based on the RNA-seq results from TCGA database of ovarian cancers using TIMER. The corrected partial Spearman's correlation coefficient and statistical *P* value were presented. **E** Representative western blot gel documents and summarized data showing the activity of ERK/AKT/NFκB p65 and C/EBPβ in TCM-educated THP1-derived macrophages stimulated with PBS. β-action was used as a loading control (n=3 independent replicates; two-way ANOVA). **F** Supernatants of THP1 cells treated with or without TCM, PXM-53, Erk inhibitor (10 µM), Akt (10 µM) inhibitor or p65 inhibitor (10 µM) were harvested for quantifying TNF-α production by ELISA assay (n=3 independent replicates; one-way ANOVA). **G** Activity of STAT1 and STAT6 in THP1 cells treated with PMX-53 or PBS were determined by Western blot analysis. β-action was used as a loading control (n=3 independent replicates; two-way ANOVA). **H** Serums of ID8 tumor-bearing WT or C5aR$^{-/-}$ mice receiving clodronate liposomes or control liposomes treatment were harvested for quantifying TNF-α production by ELISA assay (n = 3 mice per group; one-way ANOVA). **I** Proportion of CD8$^+$ T, IFN-γ$^+$CD8$^+$ T cells and PD-1 expression on CD8$^+$ T cells in ID8 tumors from WT and C5aR$^{-/-}$ mice receiving clodronate liposomes or control liposomes treatment was measured by flow cytometry (n = 3 mice; one-way ANOVA). **J** Weight of tumors on day 60 from WT and C5aR$^{-/-}$ mice receiving clodronate liposomes treatment alone or combined with PMX-53 (n = 4 mice; one-way ANOVA). Results are representative of three independent experiments. Data are represented as the mean ± SEM. *$p < 0.05$, **$p < 0.01$, ***$p < 0.001$. ****$p < 0.0001$, ns represents no significance.

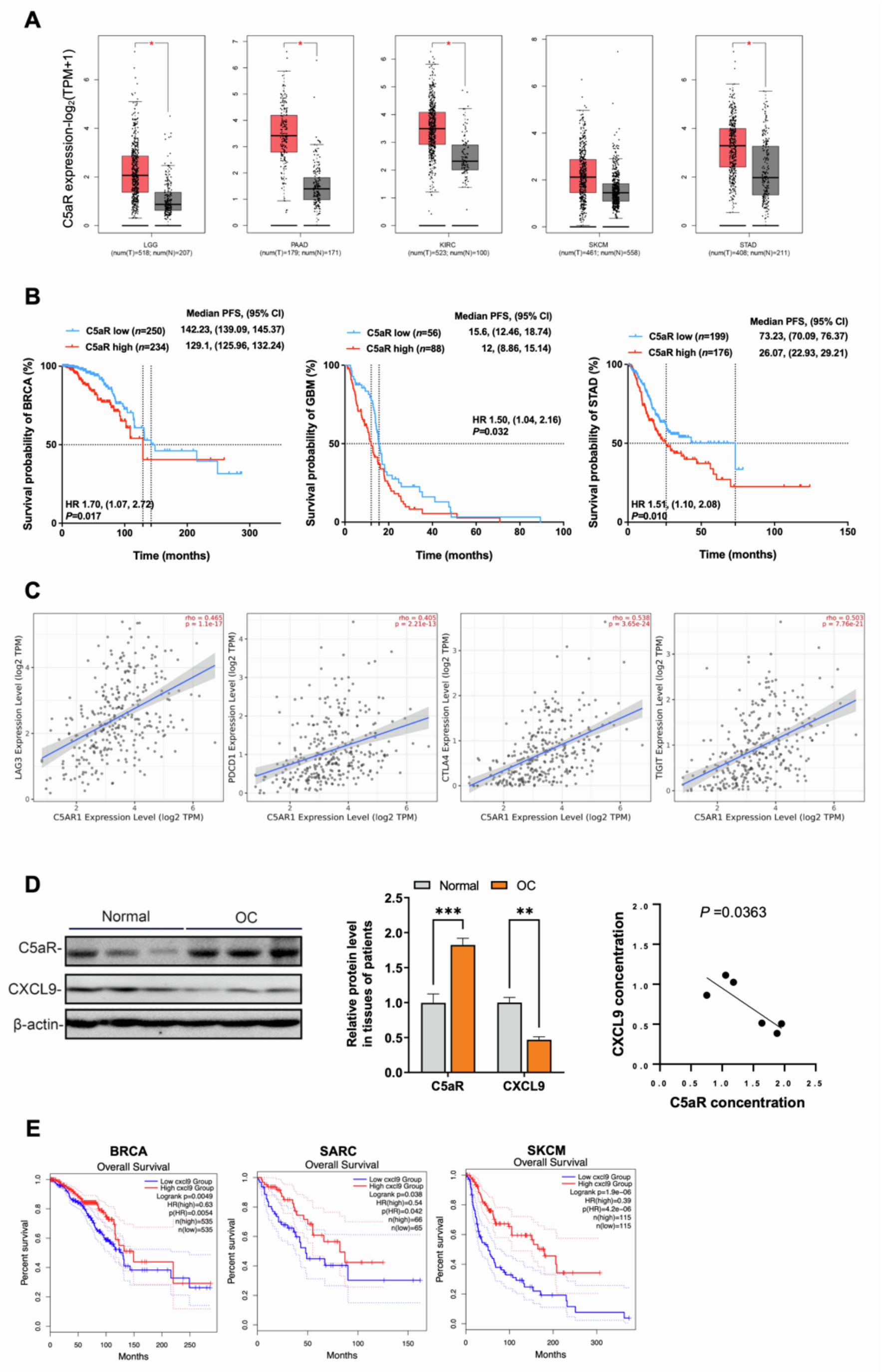

Fig.S8 C5aR links CXCL9 and CD8^{+} T cell abundance in cancer patients and involves in patients' poor outcome (related to Fig.6)

A *C5aR* expression in multiple types of tumor samples and corresponding normal samples from RNA-seq results of TCGA database using GEPIA website. LGG, Brain Lower Grade Glioma; PAAD, Pancreatic adenocarcinoma; KIRC, Kidney renal clear cell carcinoma; SKCM, Skin Cutaneous Melanoma; STAD, Stomach adenocarcinoma. Data were analyzed by two-tailed unpaired *t* test. **B** Kaplan–Meier analysis of the relationship between the *C5aR* mRNA expression and prognosis of various tumors from the TCGA dataset, C5aR low: patients with a low mRNA expression of *C5aR*, C5aR high: patients with a high mRNA expression of *C5aR*. BRCA, Breast invasive carcinoma; GBM, Glioblastoma multiforme; STAD, Stomach adenocarcinoma. Data were analyzed by log-rank test, *p* value showed the statistical difference. **C** Analysis of correlations between C5aR expression and exhausted T cell related markers (*LAG3*, *PDCD1*, *CTLA4* and *TIGIT*) based on the RNA-seq results from TCGA database of ovarian cancers using TIMER. The corrected partial Spearman's correlation coefficient and statistical *P* value were presented. **D** Expression levels of C5aR and CXCL9 protein in ovarian tissues of OC and normal patients and correlation analysis between them. (n = 3 independent replicates; two-way ANOVA). R-squared and *P* value of Pearson correlation analysis. **E** Kaplan–Meier analysis of the relationship between the *CXCL9* mRNA expression and prognosis of various tumors from the TCGA dataset. BRCA, Breast invasive carcinoma; SARC, Sarcoma; SKCM, Skin Cutaneous Melanoma. Data were analyzed by log-rank test, *p* value showed the statistical difference. Data are represented as the mean ± SEM. *$p < 0.05$, **$p < 0.01$, ***$p < 0.001$. ****$p < 0.0001$, ns represents no significance.

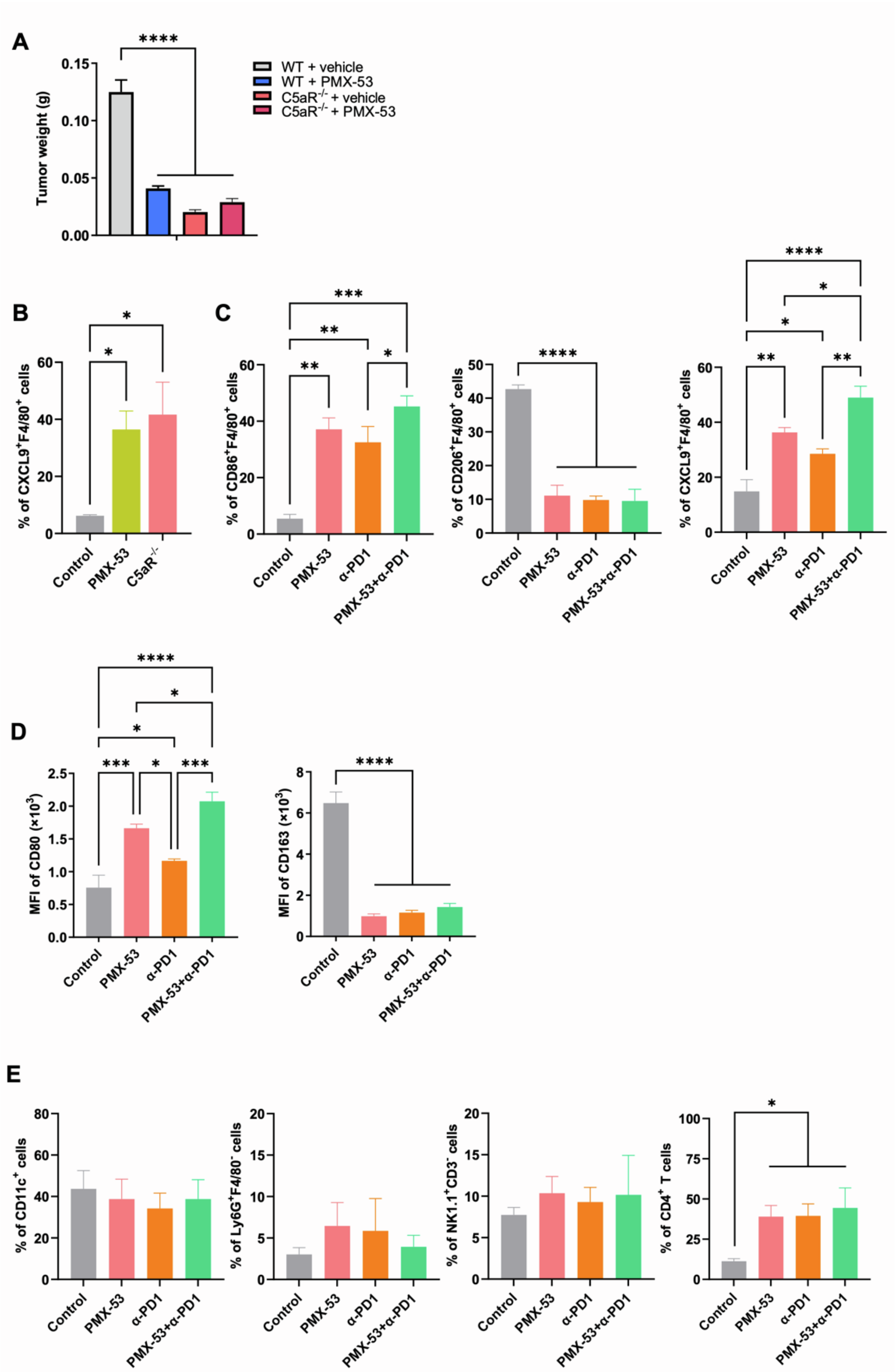

Fig.S9 C5aR antagonist and PD-1 immune checkpoint blockade therapy promotes anti-tumor immunity (related to Fig.7)

A Weight of tumors on day 60 from WT and C5aR$^{-/-}$ mice receiving PBS or PMX-53 treatment (n = 3 mice; one-way ANOVA). **B** Proportion of tumor-infiltrating CXCL9^{+}F4/80^{+} cells from WT mice receiving PMX-53 or Vehicle treatment and C5aR$^{-/-}$ mice was measured by flow cytometry (n = 3 mice; one-way ANOVA). **C** Proportion of tumor-infiltrating CD86^{+}F4/80^{+}, CD206^{+}F4/80^{+} and CXCL9^{+}F4/80^{+} cells from WT mice receiving treatment of αPD-1 or PMX-53 alone or in combination was measured by flow cytometry (n = 3 mice; one-way ANOVA). **D** Flow cytometry analysis of expression of CD80 and CD163 on F4/80^{+} TAM from WT mice receiving treatment of αPD-1 or PMX-53 alone or in combination (n = 3 mice; one-way ANOVA). **E** Proportion of tumor-infiltrating DC (CD11c^{+}), neutrophil (Ly6G^{+}F4/80^{-}), NK (NK1.1^{+}CD3^{-}) and CD4^{+} T cells from WT mice receiving treatment of αPD-1 or PMX-53 alone or in combination was measured by flow cytometry (n = 3 mice; one-way ANOVA). Results are representative of three independent experiments. Data are represented as the mean ± SEM. *$p < 0.05$, **$p < 0.01$, ***$p < 0.001$. ****$p < 0.0001$, ns represents no significance.

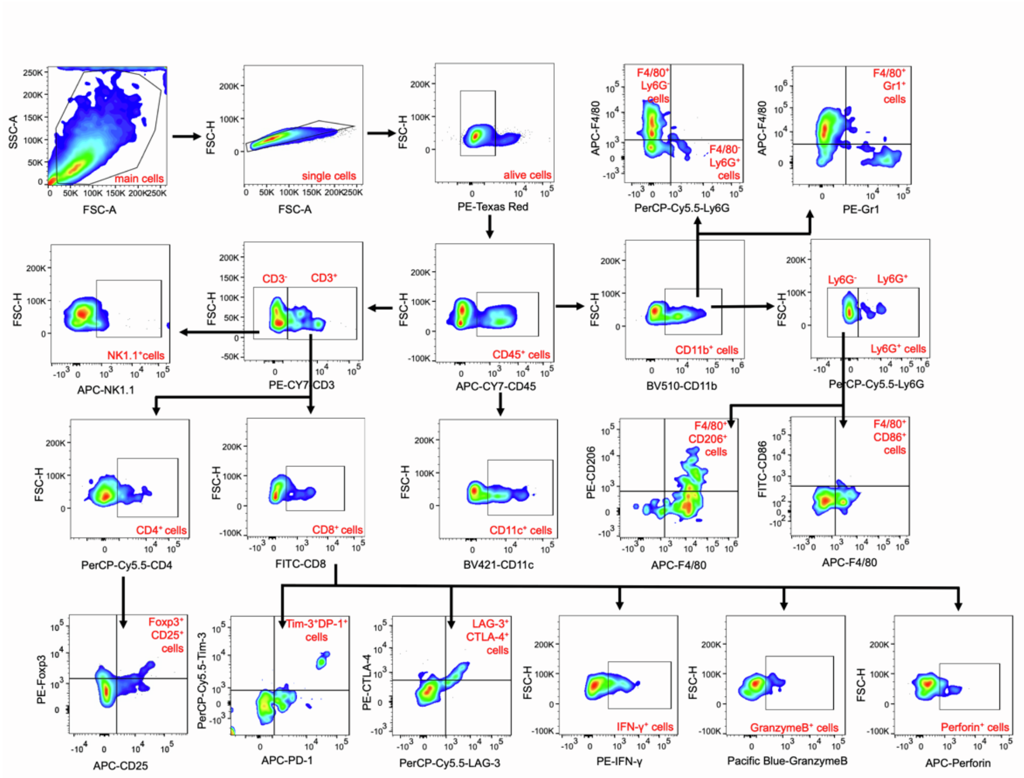

Fig.S10 Gating strategies for identifying indicated cell populations in TME (related to Fig.1, 2, 3, 5 and 7)

Table S1. Clinicopathological characteristics in ovarian cancer patients

**Characteristic**	**Cases, n (%)**
**Age (years)**	
≥55	8(53.3)
<55	7(46.7)
**FIGO stage**	
I	6(40.0)
II	5(33.3)
III	4(26.7)
IV	0
**Histological type**	
Serous adenocarcinoma	12(80.0)
Mucoid adenocarcinoma	2(13.3)
Endometrial adenocarcinoma	1(6.7)
**Intraperitoneal metastasis**	
No	11(73.3)
Yes	4(26.7)
**Intestinal metastasis**	
No	9(60.0)
Yes	6(40.0)
**Ascites**	
No	4(26.7)
Yes	11(73.3)

Table S2. Primer sequencesc

Primer	sequences
Mouse-TNFα-F	CGCTCTTCTGTCTACTGAACTTCGG
Mouse-TNFα-R	GTGGTTTGTGAGTGTGAGGGTCTG
Mouse-IL-6-F	CTTCTTGGGACTGATGCTGGTGAC
Mouse-IL-6-R	AGTGGTATCCTCTGTGAAGTCTCCTC
Mouse-IL-4-F	TACCAGGAGCCATATCCACGGATG
Mouse-IL-4-R	TGTGGTGTTCTTCGTTGCTGTGAG
Mouse-TFGβ-F	GCAACAATTCCTGGCGTTACCTTG
Mouse-TFGβ-R	GAAAGCCCTGTATTCCGTCTCCTTG
Mouse-IL-10-F	CTGGACAACATACTGCTAACCGACTC
Mouse-IL-10-R	ACTGGATCATTTCCGATAAGGCTTGG
Mouse-iNOS-F	ATCTTGGAGCGAGTTGTGGATTGTC
Mouse-iNOS-R	TCGTAATGTCCAGGAAGTAGGTGAGG
Mouse-IL-23-F	GGACTCAAGGACAACAGCCAGTTC
Mouse-IL-23-R	TGAAGATGTCAGAGTCAAGCAGGTG
Mouse-IL-12-F	CGTTTATGTTGTAGAGGTGGACTGGAC
Mouse-IL-12-R	ATGTCATCTTCTTCAGGCGTGTCAC
Mouse-PDL2-F	GCCTCAGCCTAGCAGAAACTTCAG
Mouse-PDL2-R	GACTTTGGGTTCCATCCGACTCAG
Mouse-Arg1-F	AGACAGCAGAGGAGGTGAAGAGTAC
Mouse-Arg1-R	AAGGTAGTCAGTCCCTGGCTTATGG
Mouse-FIZZ1-F	TCGTGGAGAATAAGGTCAAGGAACTTC
Mouse-FIZZ1-R	CAAGCACACCCAGTAGCAGTCATC
Mouse-CD86-F	TCTGCCGTGCCCATTTACAAAGG
Mouse-CD86-R	TGCCCAAATAGTGCTCGTACAGAAC
Mouse-CD80-F	GACCCTCCTGATAGCAAGAACACAC
Mouse-CD80-R	ATGATGACAACGATGACGACGACTG
Mouse-CD206-F	GTCTGAGTGTACGCAGTGGTTGG
Mouse-CD206-R	TCTGATGATGGACTTCCTGGTAGCC
Mouse-CCL2-F	CACTCACCTGCTGCTACTCATTCAC

Mouse-CCL2-R	CACTCACCTGCTGCTACTCATTCAC
Mouse-CCL8-F	GCTCCAGTCACCTGCTGCTTTC
Mouse-CCL8-R	ACACAGAGAGACATACCCTGCTTGG
Mouse-CCL20-F	AGGCAGAAGCAAGCAACTACGAC
Mouse-CCL20-R	ATCGGCCATCTGTCTTGTGAAACC
Mouse-CXCL12-F	CATCGCCAGAGCCAACGTCAAG
Mouse-CXCL12-R	TCGGGTCAATGCACACTTGTCTG
Mouse-CXCL9-F	CTCGGCAAATGTGAAGAAGCTGATG
Mouse-CXCL9-R	TTCCTTGAACGACGACGACTTTGG
Mouse-CCL4-F	CGTGTCTGCCCTCTCTCTCCTC
Mouse-CCL4-R	GCAGGAAGTGGGAGGGTCAGAG
Mouse-PDCD1-F	GGTATCCCTGTATTGCTGCTGCTG
Mouse-PDCD1-R	CTTCAGAGTGTCGTCCTTGCTTCC
Mouse-CTLA4-F	GCGGCAGACAAATGACCAAATGAC
Mouse-CTLA4-R	CAACAGCTCTCAGTCCTTGGATGG
Mouse-LAG3-F	GCCATCTCGTTCTCGTTCTCATCC
Mouse-LAG3-R	TTCTCCACCAGTGAAAGCCAAAGG
Mouse-GZMB-F	GTGCTGACTGCTGCTCACTGTG
Mouse-GZMB-R	TTGCTGGGTCTTCTCCTGTTCTTTG
Mouse-PRF1-F	CTCCTCCTATGGCACGCACTTTATC
Mouse-PRF1-R	TTCAGGCAGTCTCCTACCTCATCAG
Mouse-IFNG-F	CTGGAGGAACTGGCAAAAGGATGG
Mouse-IFNG-R	GACGCTTATGTTGTTGCTGATGGC
Human-C5aR-F	GGTGTTGTGTGGCGTGGACTAC
Human-C5aR-R	AACAAATCGTGAGCGTGAGTAGAGG
Human-CXCL9-F	TCTTGCTGGTTCTGATTGGAGTGC
Human-CXCL9-R	GATAGTCCCTTGGTTGGTGCTGATG

References

1. Ricklin, D., Hajishengallis, G., Yang, K., and Lambris, J. (2010). Complement: a key system for immune surveillance and homeostasis. Nature immunology *11*, 785-797. 10.1038/ni.1923.
2. Schmidt, C., Lambris, J., and Ricklin, D. (2016). Protection of host cells by complement regulators. Immunological reviews *274*, 152-171. 10.1111/imr.12475.
3. Leslie, M. (2012). Immunology. The new view of complement. Science (New York, N.Y.) *337*, 1034-1037. 10.1126/science.337.6098.1034.
4. Kolev, M., Le Friec, G., and Kemper, C. (2014). Complement--tapping into new sites and effector systems. Nature reviews. Immunology *14*, 811-820. 10.1038/nri3761.
5. Nitta, H., Wada, Y., Kawano, Y., Murakami, Y., Irie, A., Taniguchi, K., Kikuchi, K., Yamada, G., Suzuki, K., Honda, J., et al. (2013). Enhancement of human cancer cell motility and invasiveness by anaphylatoxin C5a via aberrantly expressed C5a receptor (CD88). Clinical cancer research : an official journal of the American Association for Cancer Research *19*, 2004-2013. 10.1158/1078-0432.Ccr-12-1204.
6. Chen, J., Li, G., Zhang, L., Tang, M., Cao, X., Xu, G., and Wu, Y. (2018). Complement C5a/C5aR pathway potentiates the pathogenesis of gastric cancer by down-regulating p21 expression. Cancer letters *412*, 30-36. 10.1016/j.canlet.2017.10.003.
7. Imamura, T., Yamamoto-Ibusuki, M., Sueta, A., Kubo, T., Irie, A., Kikuchi, K., Kariu, T., and Iwase, H. (2016). Influence of the C5a-C5a receptor system on breast cancer progression and patient prognosis. Breast cancer (Tokyo, Japan) *23*, 876-885. 10.1007/s12282-015-0654-3.
8. Saito, K., Iioka, H., Maruyama, S., Sumardika, I., Sakaguchi, M., and Kondo, E. (2019). PODXL1 promotes metastasis of the pancreatic ductal adenocarcinoma by activating the C5aR/C5a axis from the tumor microenvironment. Neoplasia (New York, N.Y.) *21*, 1121-1132. 10.1016/j.neo.2019.09.003.
9. Nunez-Cruz, S., Gimotty, P., Guerra, M., Connolly, D., Wu, Y., DeAngelis, R., Lambris, J., Coukos, G., and Scholler, N. (2012). Genetic and pharmacologic inhibition of complement impairs endothelial cell function and ablates ovarian cancer neovascularization. Neoplasia (New York, N.Y.) *14*, 994-1004. 10.1593/neo.121262.
10. Ding, P., Li, L., Li, L., Lv, X., Zhou, D., Wang, Q., Chen, J., Yang, C., Xu, E., Dai, W., et al. (2020). C5aR1 is a master regulator in Colorectal Tumorigenesis via Immune modulation. Theranostics *10*, 8619-8632. 10.7150/thno.45058.
11. Markiewski, M., DeAngelis, R., Benencia, F., Ricklin-Lichtsteiner, S., Koutoulaki, A., Gerard, C., Coukos, G., and Lambris, J. (2008). Modulation of the antitumor immune response by complement. Nature immunology *9*, 1225-1235. 10.1038/ni.1655.
12. Vadrevu, S., Chintala, N., Sharma, S., Sharma, P., Cleveland, C., Riediger, L., Manne, S., Fairlie, D., Gorczyca, W.,

Almanza, O., et al. (2014). Complement c5a receptor facilitates cancer metastasis by altering T-cell responses in the metastatic niche. Cancer research *74*, 3454-3465. 10.1158/0008-5472.Can-14-0157.

13. Janelle, V., and Lamarre, A. (2014). Role of the complement system in NK cell-mediated antitumor T-cell responses. Oncoimmunology *3*, e27897. 10.4161/onci.27897.

14. Wang, Y., Sun, S., Liu, Q., Yu, Y., Guo, J., Wang, K., Xing, B., Zheng, Q., Campa, M., Patz, E., et al. (2016). Autocrine Complement Inhibits IL10-Dependent T-cell-Mediated Antitumor Immunity to Promote Tumor Progression. Cancer discovery *6*, 1022-1035. 10.1158/2159-8290.Cd-15-1412.

15. Corrales, L., Ajona, D., Rafail, S., Lasarte, J., Riezu-Boj, J., Lambris, J., Rouzaut, A., Pajares, M., Montuenga, L., and Pio, R. (2012). Anaphylatoxin C5a creates a favorable microenvironment for lung cancer progression. Journal of immunology (Baltimore, Md. : 1950) *189*, 4674-4683. 10.4049/jimmunol.1201654.

16. Gunn, L., Ding, C., Liu, M., Ma, Y., Qi, C., Cai, Y., Hu, X., Aggarwal, D., Zhang, H., and Yan, J. (2012). Opposing roles for complement component C5a in tumor progression and the tumor microenvironment. Journal of immunology (Baltimore, Md. : 1950) *189*, 2985-2994. 10.4049/jimmunol.1200846.

17. Pollard, J. (2004). Tumour-educated macrophages promote tumour progression and metastasis. Nature reviews. Cancer *4*, 71-78. 10.1038/nrc1256.

18. Pyonteck, S., Akkari, L., Schuhmacher, A., Bowman, R., Sevenich, L., Quail, D., Olson, O., Quick, M., Huse, J., Teijeiro, V., et al. (2013). CSF-1R inhibition alters macrophage polarization and blocks glioma progression. Nature medicine *19*, 1264-1272. 10.1038/nm.3337.

19. Ostuni, R., Kratochvill, F., Murray, P., and Natoli, G. (2015). Macrophages and cancer: from mechanisms to therapeutic implications. Trends in immunology *36*, 229-239. 10.1016/j.it.2015.02.004.

20. Kurahara, H., Shinchi, H., Mataki, Y., Maemura, K., Noma, H., Kubo, F., Sakoda, M., Ueno, S., Natsugoe, S., and Takao, S. (2011). Significance of M2-polarized tumor-associated macrophage in pancreatic cancer. The Journal of surgical research *167*, e211-219. 10.1016/j.jss.2009.05.026.

21. Shabo, I., Stål, O., Olsson, H., Doré, S., and Svanvik, J. (2008). Breast cancer expression of CD163, a macrophage scavenger receptor, is related to early distant recurrence and reduced patient survival. International journal of cancer *123*, 780-786. 10.1002/ijc.23527.

22. Lee, C., Espinosa, I., Vrijaldenhoven, S., Subramanian, S., Montgomery, K., Zhu, S., Marinelli, R., Peterse, J., Poulin, N., Nielsen, T., et al. (2008). Prognostic significance of macrophage infiltration in leiomyosarcomas. Clinical cancer research : an official journal of the American Association for Cancer Research *14*, 1423-1430. 10.1158/1078-0432.Ccr-07-1712.

23. Medler, T., Murugan, D., Horton, W., Kumar, S., Cotechini, T., Forsyth, A., Leyshock, P., Leitenberger, J., Kulesz-

Martin, M., Margolin, A., et al. (2018). Complement C5a Fosters Squamous Carcinogenesis and Limits T Cell Response to Chemotherapy. Cancer cell *34*, 561-578.e566. 10.1016/j.ccell.2018.09.003.

24. Piao, C., Cai, L., Qiu, S., Jia, L., Song, W., and Du, J. (2015). Complement 5a Enhances Hepatic Metastases of Colon Cancer via Monocyte Chemoattractant Protein-1-mediated Inflammatory Cell Infiltration. The Journal of biological chemistry *290*, 10667-10676. 10.1074/jbc.M114.612622.

25. Piao, C., Zhang, W., Li, T., Zhang, C., Qiu, S., Liu, Y., Liu, S., Jin, M., Jia, L., Song, W., and Du, J. (2018). Complement 5a stimulates macrophage polarization and contributes to tumor metastases of colon cancer. Experimental cell research *366*, 127-138. 10.1016/j.yexcr.2018.03.009.

26. Li, T., Fan, J., Wang, B., Traugh, N., Chen, Q., Liu, J., Li, B., and Liu, X. (2017). TIMER: A Web Server for Comprehensive Analysis of Tumor-Infiltrating Immune Cells. Cancer research *77*, e108-e110. 10.1158/0008-5472.Can-17-0307.

27. Martinez, F., Gordon, S., Locati, M., and Mantovani, A. (2006). Transcriptional profiling of the human monocyte-to-macrophage differentiation and polarization: new molecules and patterns of gene expression. Journal of immunology (Baltimore, Md. : 1950) *177*, 7303-7311. 10.4049/jimmunol.177.10.7303.

28. Ruffell, B., Chang-Strachan, D., Chan, V., Rosenbusch, A., Ho, C.M.T., Pryer, N., Daniel, D., Hwang, E.S., Rugo, H.S., and Coussens, L.M. (2014). Macrophage IL-10 blocks CD8+ T cell-dependent responses to chemotherapy by suppressing IL-12 expression in intratumoral dendritic cells. Cancer Cell *26*, 623-637. 10.1016/j.ccell.2014.09.006.

29. Funes, S.C., Rios, M., Escobar-Vera, J., and Kalergis, A.M. (2018). Implications of macrophage polarization in autoimmunity. Immunology *154*, 186-195. 10.1111/imm.12910.

30. Chevrier, S., Levine, J.H., Zanotelli, V.R.T., Silina, K., Schulz, D., Bacac, M., Ries, C.H., Ailles, L., Jewett, M.A.S., Moch, H., et al. (2017). An Immune Atlas of Clear Cell Renal Cell Carcinoma. Cell *169*. 10.1016/j.cell.2017.04.016.

31. Finch, A., Wong, A., Paczkowski, N., Wadi, S., Craik, D., Fairlie, D., and Taylor, S. (1999). Low-molecular-weight peptidic and cyclic antagonists of the receptor for the complement factor C5a. Journal of medicinal chemistry *42*, 1965-1974. 10.1021/jm9806594.

32. Jiang, P., Gu, S., Pan, D., Fu, J., Sahu, A., Hu, X., Li, Z., Traugh, N., Bu, X., Li, B., et al. (2018). Signatures of T cell dysfunction and exclusion predict cancer immunotherapy response. Nature medicine *24*, 1550-1558. 10.1038/s41591-018-0136-1.

33. Wherry, E., and Kurachi, M. (2015). Molecular and cellular insights into T cell exhaustion. Nature reviews. Immunology *15*, 486-499. 10.1038/nri3862.

34. Tang, Z., Li, C., Kang, B., Gao, G., Li, C., and Zhang, Z. (2017). GEPIA: a web server for cancer and normal gene expression profiling and interactive analyses. Nucleic acids research *45*, W98-W102. 10.1093/nar/gkx247.

35. Ding, Q., Lu, P., Xia, Y., Ding, S., Fan, Y., Li, X., Han, P., Liu, J., Tian, D., and Liu, M. (2016). CXCL9: evidence and contradictions for its role in tumor progression. Cancer medicine *5*, 3246-3259. 10.1002/cam4.934.

36. Ben-Neriah, Y., and Karin, M. (2011). Inflammation meets cancer, with NF-κB as the matchmaker. Nature immunology *12*, 715-723. 10.1038/ni.2060.

37. Poli, V. (1998). The role of C/EBP isoforms in the control of inflammatory and native immunity functions. The Journal of biological chemistry *273*, 29279-29282. 10.1074/jbc.273.45.29279.

38. van Rooijen, N., Kors, N., ter Hart, H., and Claassen, E. (1988). In vitro and in vivo elimination of macrophage tumor cells using liposome-encapsulated dichloromethylene diphosphonate. Virchows Archiv. B, Cell pathology including molecular pathology *54*, 241-245. 10.1007/bf02899217.

39. Woehrl, B., Brouwer, M., Murr, C., Heckenberg, S., Baas, F., Pfister, H., Zwinderman, A., Morgan, B., Barnum, S., van der Ende, A., et al. (2011). Complement component 5 contributes to poor disease outcome in humans and mice with pneumococcal meningitis. The Journal of clinical investigation *121*, 3943-3953. 10.1172/jci57522.

40. House, I., Savas, P., Lai, J., Chen, A., Oliver, A., Teo, Z., Todd, K., Henderson, M., Giuffrida, L., Petley, E., et al. (2020). Macrophage-Derived CXCL9 and CXCL10 Are Required for Antitumor Immune Responses Following Immune Checkpoint Blockade. Clinical cancer research : an official journal of the American Association for Cancer Research *26*, 487-504. 10.1158/1078-0432.Ccr-19-1868.

41. Reis, E., Mastellos, D., Ricklin, D., Mantovani, A., and Lambris, J. (2018). Complement in cancer: untangling an intricate relationship. Nature reviews. Immunology *18*, 5-18. 10.1038/nri.2017.97.

42. Roumenina, L., Daugan, M., Petitprez, F., Sautès-Fridman, C., and Fridman, W. (2019). Context-dependent roles of complement in cancer. Nature reviews. Cancer *19*, 698-715. 10.1038/s41568-019-0210-0.

43. Zha, H., Han, X., Zhu, Y., Yang, F., Li, Y., Li, Q., Guo, B., and Zhu, B. (2017). Blocking C5aR signaling promotes the anti-tumor efficacy of PD-1/PD-L1 blockade. Oncoimmunology *6*, e1349587. 10.1080/2162402x.2017.1349587.

44. Dunkelberger, J., Zhou, L., Miwa, T., and Song, W. (2012). C5aR expression in a novel GFP reporter gene knockin mouse: implications for the mechanism of action of C5aR signaling in T cell immunity. Journal of immunology (Baltimore, Md. : 1950) *188*, 4032-4042. 10.4049/jimmunol.1103141.

45. Reichhardt, M., and Meri, S. (2018). Intracellular complement activation-An alarm raising mechanism? Seminars in immunology *38*, 54-62. 10.1016/j.smim.2018.03.003.

46. Verschoor, A., Karsten, C., Broadley, S., Laumonnier, Y., and Köhl, J. (2016). Old dogs-new tricks: immunoregulatory properties of C3 and C5 cleavage fragments. Immunological reviews *274*, 112-126. 10.1111/imr.12473.

47. Xi, W., Liu, L., Wang, J., Xia, Y., Bai, Q., Xiong, Y., Qu, Y., Long, Q., Xu, J., and Guo, J. (2016). Enrichment of C5a-C5aR axis predicts poor postoperative prognosis of patients with clear cell renal cell carcinoma. Oncotarget *7*, 80925-

80934. 10.18632/oncotarget.13108.

48. Magrini, E., Di Marco, S., Mapelli, S., Perucchini, C., Pasqualini, F., Donato, A., Guevara Lopez, M., Carriero, R., Ponzetta, A., Colombo, P., et al. (2021). Complement activation promoted by the lectin pathway mediates C3aR-dependent sarcoma progression and immunosuppression. Nature cancer *2*, 218-232. 10.1038/s43018-021-00173-0.

49. Galon, J., Costes, A., Sanchez-Cabo, F., Kirilovsky, A., Mlecnik, B., Lagorce-Pagès, C., Tosolini, M., Camus, M., Berger, A., Wind, P., et al. (2006). Type, density, and location of immune cells within human colorectal tumors predict clinical outcome. Science (New York, N.Y.) *313*, 1960-1964. 10.1126/science.1129139.

50. Gajewski, T., Schreiber, H., and Fu, Y. (2013). Innate and adaptive immune cells in the tumor microenvironment. Nature immunology *14*, 1014-1022. 10.1038/ni.2703.

51. Moser, B., Wolf, M., Walz, A., and Loetscher, P. (2004). Chemokines: multiple levels of leukocyte migration control. Trends in immunology *25*, 75-84. 10.1016/j.it.2003.12.005.

52. Harlin, H., Meng, Y., Peterson, A., Zha, Y., Tretiakova, M., Slingluff, C., McKee, M., and Gajewski, T. (2009). Chemokine expression in melanoma metastases associated with CD8+ T-cell recruitment. Cancer research *69*, 3077-3085. 10.1158/0008-5472.Can-08-2281.

53. Denkert, C., von Minckwitz, G., Brase, J., Sinn, B., Gade, S., Kronenwett, R., Pfitzner, B., Salat, C., Loi, S., Schmitt, W., et al. (2015). Tumor-infiltrating lymphocytes and response to neoadjuvant chemotherapy with or without carboplatin in human epidermal growth factor receptor 2-positive and triple-negative primary breast cancers. Journal of clinical oncology : official journal of the American Society of Clinical Oncology *33*, 983-991. 10.1200/jco.2014.58.1967.

54. Pascual-García, M., Bonfill-Teixidor, E., Planas-Rigol, E., Rubio-Perez, C., Iurlaro, R., Arias, A., Cuartas, I., Sala-Hojman, A., Escudero, L., Martínez-Ricarte, F., et al. (2019). LIF regulates CXCL9 in tumor-associated macrophages and prevents CD8 T cell tumor-infiltration impairing anti-PD1 therapy. Nature communications *10*, 2416. 10.1038/s41467-019-10369-9.

55. Czystowska-Kuzmicz, M., Sosnowska, A., Nowis, D., Ramji, K., Szajnik, M., Chlebowska-Tuz, J., Wolinska, E., Gaj, P., Grazul, M., Pilch, Z., et al. (2019). Small extracellular vesicles containing arginase-1 suppress T-cell responses and promote tumor growth in ovarian carcinoma. Nature communications *10*, 3000. 10.1038/s41467-019-10979-3.

56. Yang, X., Lin, J., Wang, G., and Xu, D. (2022). Targeting Proliferating Tumor-Infiltrating Macrophages Facilitates Spatial Redistribution of CD8 T Cells in Pancreatic Cancer. Cancers *14*. 10.3390/cancers14061474.

57. Luan, X., Yan, Y., Zheng, Q., Wang, M., Chen, W., Yu, J., and Fang, J. (2020). Excessive reactive oxygen species induce apoptosis via the APPL1-Nrf2/HO-1 antioxidant signalling pathway in trophoblasts with missed abortion. Life sciences *254*, 117781. 10.1016/j.lfs.2020.117781.
